# Development of a Traditional Chinese Medicine Lifestyle Medicine Program for Depression: A Multi-Method Study

**DOI:** 10.3390/healthcare14121631

**Published:** 2026-06-09

**Authors:** Jia Yin Ruan, Sha Li, Fen Xu, Fiona Yan Yee Ho, Teris Cheung, Janice Yuen Shan Ho, Wai Chi Chan, Hai Yong Chen, Dennis Cheuk Wing Au, Rebecca Wing Yan Lee, Yim Wah Mak, Wing Fai Yeung

**Affiliations:** 1 School of Nursing, The Hong Kong Polytechnic University Hung Hom, Kowloon, Hong Kong, China; jr7121@nyu.edu (J.Y.R.); teris.cheung@polyu.edu.hk (T.C.); janice.ys.ho@polyu.edu.hk (J.Y.S.H.); yw.mak@polyu.edu.hk (Y.W.M.); 2 Rory Meyers College of Nursing, New York University, New York, NY 10010, USA; 3 School of Nursing, Nanjing Medical University, Nanjing 211166, China; shali@njmu.edu.cn; 4 Department of Nursing Science, Faculty of Medicine, University of Malaya, Kuala Lumpur 50603, Malaysia; 22075483@siswa.um.edu.my; 5 Department of Psychology, The Chinese University of Hong Kong, Hong Kong, China; fionahoyy@cuhk.edu.hk; 6 Department of Psychiatry, The University of Hong Kong, Hong Kong, China; waicchan@hku.hk; 7 School of Chinese Medicine, The University of Hong Kong, Hong Kong, China; haiyong@hku.hk; 8 Hong Kong Association of Traditional Chinese Medicine Ltd., Hong Kong Island, Hong Kong, China; dennis@cmedforall.org; 9 Sin-Hua Herbalists’ and Herb Dealers’ Promotion Society Limited, Hong Kong, China; sinhuahhd12@yahoo.com.hk; 10 Research Centre for Chinese Medicine Innovation, The Hong Kong Polytechnic University, Hong Kong, China

**Keywords:** Delphi technique, depression, lifestyle, program development, traditional Chinese medicine

## Abstract

**Introduction**: Evidence supports using multicomponent lifestyle medicine programs to alleviate depression, yet few studies detail the program development process. This study aimed to systematically develop a complex lifestyle medicine program for depression based on Traditional Chinese Medicine theory. **Methods:** A stepwise, multi-method study was conducted. The preparation phase involved understanding the public health issue, identifying resources, and reviewing published evidence. Based on these results, a Delphi survey was performed, followed by an analysis of the context understanding as well as design and refinement of the program. Subsequently, the program was modeled, and a program theory was developed, incorporating explanations and assumptions in a relevant conceptual framework and logic model. **Results:** A complex lifestyle medicine program and program manual were established. The program involves three major themes, each with six 120 min weekly consecutive sessions: *nourishing the heart* (two sessions), *nourishing according to the time* (two sessions), and *nourishing the Qi* (two sessions). The program covered TCM theory and practice to promote healthy sleep, diet, stress management, and self-administered acupressure and TCM exercise. Teaching materials were created accordingly. **Conclusions:** This study reports the development of a complex multicomponent TCM lifestyle medicine program to relieve depression thoroughly and transparently, with a specific focus on the Hong Kong context and a particular focus on TCM theory. The developed program will be examined for feasibility, acceptability, and preliminary efficacy in alleviating depression in a mixed-methods clinical study.

## 1. Introduction

Depression is a prevalent and debilitating mental disorder characterized by persistent low mood (e.g., sadness, irritability, or emptiness), accompanied by cognitive and somatic disturbances that substantially impair daily functioning [1]. It is a major contributor to disability, reduced quality of life, and economic burden at both individual and societal levels [2]. Globally, the prevalence of depression has increased markedly over recent decades, rising by 88.52% between 1990 and 2021 [3]. As a leading contributor to the global burden of disease, depression affected an estimated 332.41 million people worldwide [4] and is projected to cost the global economy US$16 trillion by 2030 [5]. Despite the availability of treatments, including pharmacotherapy (e.g., selective serotonin reuptake inhibitors) and psychotherapy (e.g., cognitive behavioral therapy), depression remains insufficiently managed at the population level [6]. This persistent burden has been attributed to multiple factors, including limited help-seeking behaviors [7], inequitable access to care [8], suboptimal treatment quality [9], poor adherence [10], and incomplete or unsustained treatment response [11]. These limitations highlight the need for complementary approaches that are accessible, acceptable, culturally sensitive, and capable of addressing modifiable risk factors. In this context, lifestyle medicine—defined as the use of evidence-based lifestyle interventions (e.g., diet, physical activity, sleep, stress management, and social connection) to prevent and manage disease—has emerged as a promising adjunctive strategy for depression.

Multicomponent lifestyle medicine interventions, which simultaneously target multiple behavioral domains such as diet, sleep hygiene, physical activity, and stress management (e.g., mindfulness and meditation), have demonstrated beneficial effects on depressive symptoms [12,13,14,15,16,17,18]. However, the implementation and sustainability of lifestyle changes are inherently complex, shaped by multilevel influences including culture, socioeconomic conditions, belief systems, and local health practices [19]. This complexity underscores the importance of culturally grounded intervention frameworks.

Lifestyle medicine is typically defined as the use of evidence-based behavioral interventions to prevent and manage disease, focusing on modifiable factors such as diet, physical activity, sleep, stress, and social relationships [20,21]. In contrast, Traditional Chinese Medicine (TCM) health preservation (*yangsheng*) represents a holistic health system rooted in concepts such as balance, the regulation of *Qi*, and the integration of body and mind, emphasizing harmony between individuals and their environment [22]. Despite differences in theoretical foundations—Western lifestyle medicine being primarily behavior-focused and grounded in biomedical evidence, and TCM being system-oriented and theory-driven—there is substantial conceptual overlap between the two paradigms. Both emphasize preventive care, the central role of daily habits, and the importance of self-regulation in maintaining health [20,21,22]. This overlap provides a conceptual bridge for integrating lifestyle interventions within a TCM theoretical framework. Importantly, grounding lifestyle interventions in TCM theory may enhance cultural relevance, acceptability, and adherence in Chinese populations. In Hong Kong, TCM is deeply embedded in the health care system and everyday health practices [23], and individuals with depression are more likely to seek complementary and alternative therapies [24]. Therefore, a TCM-informed lifestyle approach may represent a culturally appropriate and context-sensitive strategy for depression management.

Emerging evidence supports the potential of TCM lifestyle approaches for depression. For instance, a meta-analysis of 18 randomized controlled trials reported improvements in depressive symptoms. However, the overall quality of evidence remains limited [25]. Importantly, most studies lacked transparency in intervention reporting—17 of the included trials did not adequately describe how intervention components were selected, developed, or implemented—thereby hindering reproducibility, scalability, and clinical translation.

Despite growing interest in TCM-based approaches, there is currently a lack of systematically developed, theory-informed, and transparently reported TCM lifestyle interventions for depression. This gap limits the reproducibility, implementation, and translation of existing evidence into practice. Developing such interventions is inherently complex and requires rigorous methodological guidance. The Medical Research Council (MRC) framework for complex interventions provides a structured approach, emphasizing theory development, evidence synthesis, stakeholder involvement, and iterative refinement. Therefore, the aim of this study is to systematically develop a multicomponent TCM-informed lifestyle medicine intervention for depression using a multi-method approach, including evidence synthesis, Delphi consensus, stakeholder engagement, and integration of cultural and contextual factors. This work aims to establish a robust foundation for subsequent feasibility testing and evaluation of intervention effectiveness.

## 2. Methods

A multi-method study was conducted to address the development phase of this complex program. In this study, “multi-method” refers to the use of more than one method within a single methodological paradigm or study design to examine related aspects of the same research problem and to generate complementary evidence from multiple perspectives, rather than to formally integrate qualitative and quantitative data as in mixed-methods research [26]. This approach was appropriate because the development of a complex program requires the use of diverse sources of evidence, including existing literature, stakeholder perspectives, contextual assessment, theory, and iterative refinement.

The study was guided by the updated MRC framework for developing and evaluating complex interventions and by published guidance on complex intervention development [27]. These sources emphasize several core tasks during intervention development, including understanding the context, engaging stakeholders, drawing on existing evidence and theory, developing or refining program theory, identifying uncertainties, and modeling how the intervention is expected to work [27,28]. Accordingly, the three-step structure used in this study was not intended to represent a universal standard or a fixed sequence prescribed by the framework. Rather, it was developed as an operational structure to organize these recommended development activities in a transparent and logical manner [29]. A similar stepwise approach has also been reported in a previous multi-method study on complex intervention development [28].

**Figure 1:**
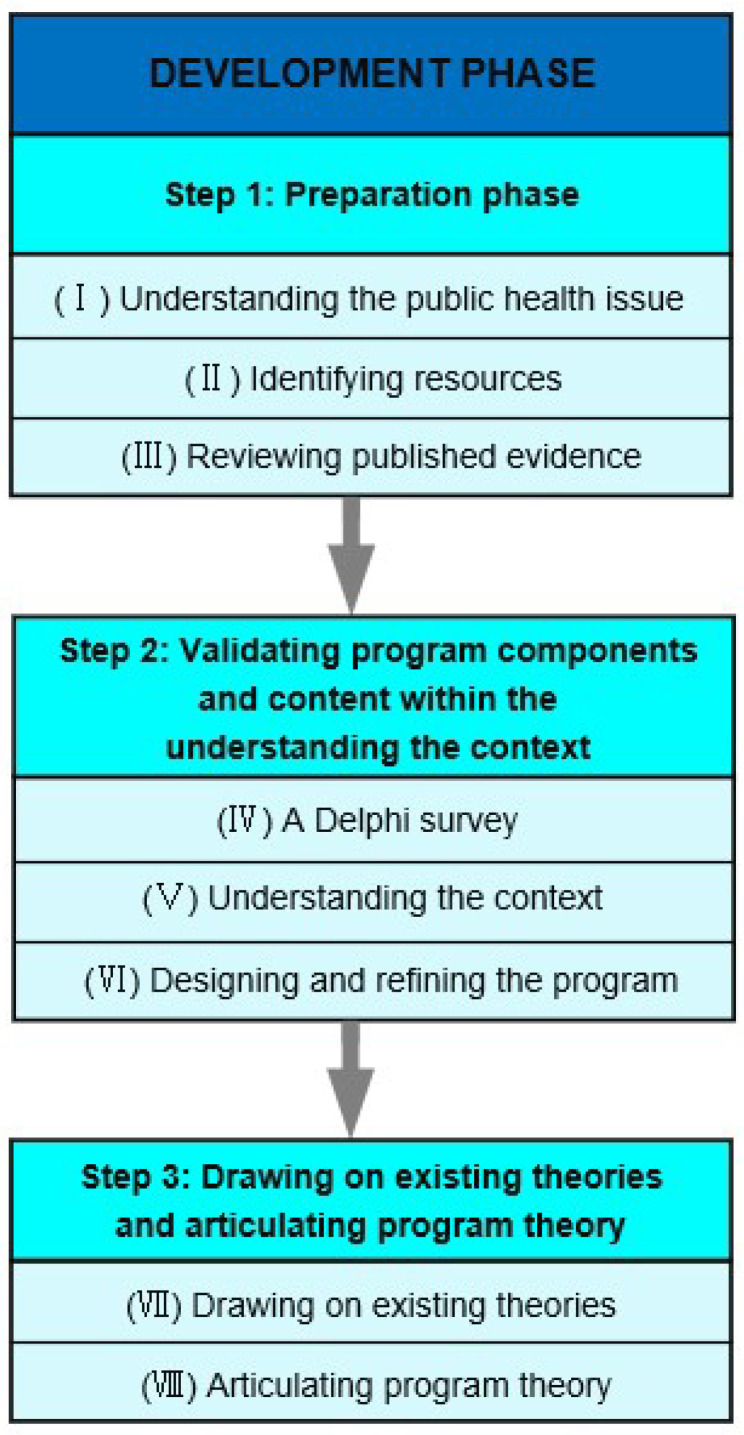
A summary of the development phase with relevant approaches and aims.

Specifically, Step 1, the preparation phase, focused on understanding the current context and practice through literature review and primary data collection with relevant stakeholders. Step 2 focused on developing and refining the intervention components and implementation strategies by integrating empirical findings, stakeholder input, and established theories. Step 3 focused on modeling the program process and expected outcomes through articulation of the program theory and development of a logic model. Figure 1 summarizes the development phase, including the methods, objectives, and outputs of each step.

### 2.1. Methods—Step 1: Preparation Phase

Step 1 is the preparation phase, which involves planning the development process. The aim of this phase was to establish and verify the urgent need for a multicomponent TCM lifestyle medicine program and to identify evidence-based content for the program. This phase consists of three parts: understanding the public health issue (depression), identifying resources, and reviewing published evidence.

#### 2.1.1. Understanding the Public Health Issue and Identifying Resources

Regarding understanding the public health issue of depression, various aspects were researched, reviewed, and analyzed. These aspects include the definition of depression [1,6], its incidence and prevalence [30], and the burden of depression [2]. Additionally, the current state of depression treatment, including unsatisfactory treatment status and the rationale behind it [7,8,9,11], highlighted the demand for innovative treatment options and preferences for complementary and alternative therapies among individuals with depression. The connection between lifestyle and lifestyle medicine for depression [31,32], and similarity between lifestyle medicine for depression and lifestyle medicine for depression based on TCM [12,13,14,15,16,17,18] were also explored.

#### 2.1.2. Reviewing Published Evidence

Reviewing published evidence involves three components: RCTs on TCM lifestyle medicine for depression [25], textbooks on TCM health preservation [22,33], and TCM clinical practice guidelines for depression treatment [34].

Notably, in order to exhaust the current available evidence regarding TCM lifestyle medicine for depression, a systematic review and meta-analysis of multicomponent health interventions based on TCM theory for depressive symptoms was performed [25]. In this systematic review, 10 English and 7 Chinese databases were searched from inception through June 2022. To contextualize the original search date and address newly published evidence, a bridging search was conducted from July 2022 to 17 May 2026, using the same search strategy and eligibility criteria. No additional eligible studies meeting the inclusion criteria were identified. Thus, the original 18 RCTs remained the evidence base for informing the development of the multicomponent lifestyle interventions based on TCM theory for depressive symptoms. These RCTs examined interventions with components including TCM exercise, depression and/or mental health education, nutritional advice and/or food therapy, sleep–wake management, psychological management, and meridian and collateral management [25]. All six components were based on TCM [25]. The information documented in previous guidelines, textbooks, and the systematic review was extracted to prepare the questionnaire for the Delphi survey in Step 2.

As the initial 61-item questionnaire was designed as a Delphi consultation tool rather than a psychometric scale, conventional reliability and validity testing was not applicable at this stage. Instead, the content validity of the questionnaires was supported through a rigorous, evidence-informed item-generation process.

### 2.2. Methods—Step 2: Validating Program Components and Content Within the Understanding the Context

The aim of step 2 was to validate program components and content within the understanding of the research context. That phase incorporated three program development actions, specifically, undertaking primary data collection (a Delphi survey), understanding the context, and designing and refining the program [35].

#### 2.2.1. Methods of Delphi Survey

##### Study Design

A modified Delphi method was adopted to build consensus on a complex TCM lifestyle medicine program for depression [36,37]. The design of the Delphi survey is outlined in Supplementary Figure S1. To ensure methodological rigor in the implementation of the Delphi procedure, this study was guided by the Guidance on Conducting and Reporting Delphi studies (CREDES) [36]. Detailed information corresponding to the CREDES checklist is presented in Supplementary Table S1.

##### Study Population, Recruitment and Sampling

Purposive sampling was adopted to recruit experts from 25 August 2022 to 1 September 2022, via email, based on the following criteria [37]: (1) having knowledge of TCM lifestyle medicine for depression, (2) having at least five years of clinical experience as a registered Chinese medicine practitioner in Hong Kong, (3) having a postgraduate degree, and (4) willing to participate. Quasi-anonymity, regarding the anonymity of the opinions and judgments of experts, was strictly maintained during the survey.

The determination of the panel size in Delphi studies is generally empirical and pragmatic rather than based on conventional statistical power calculations. It is influenced by the study purpose, Delphi design, and panel homogeneity or heterogeneity [38,39]. Methodological guidance suggests that, for relatively homogeneous expert panels, particularly in program development, 10–15 participants may be sufficient to generate meaningful and stable findings [39]. In this study, the expert panel was relatively homogeneous. Therefore, a panel size of 10 was considered appropriate for the purpose and scope of this Delphi study.

##### Data Collection and Procedure

A three-round Delphi survey was conducted through the online survey platform Qualtrics until consensus was reached. Experts in each round were provided with two weeks to respond. A stricter criterion of ≥80% of the panel selecting 6 or 7 (“agree” or “strongly agree”, and “important” or “very important”) on a 7-point Likert scale was adopted [40].

Round 1

The survey link for the demographics and the initial 61 items developed in Step 1 was sent to the 10 Delphi panel experts via email.

Round 2

This round included 19 items from the first round that received less than 80% agreement (5 revised items) and 3 new items based on expert feedback. Three themes were generated: *Nourishing the heart* (Theme 1, 14 items), *Nourishing according to time* (Theme 2, 5 items), and *Nourishing the Qi* (Theme 3, 3 items). Open-ended questions were added to gather reasoning for ratings and additional suggestions.

Round 3

Experts rated 50 items across three themes: *Nourishing the Heart* (28 items), *Nourishing According to Time* (11 items), and *Nourishing the Qi* (11 items). Open-ended questions sought further suggestions for the multicomponent program. Items were ranked, with the top 50% selected as program content, and priority was assigned to the four program dimensions, scored as follows: 4 points for 1st place, 3 points for 2nd, 2 points for 3rd, and 1 point for 4th [41].

##### Strategies to Enhance Response Rate

To increase the response rate, several strategies were implemented. First, only experts who actively responded to the pre-Delphi invitation were invited to participate in the survey [37]. Second, the research aims and Delphi method were reiterated via email in each round to reinforce the importance of active participation. Third, a research assistant monitored the responses of the Delphi panel experts [37]. Finally, to maintain interest and motivation, experts were promptly updated on the findings from each round of the Delphi survey [37].

##### Data Analysis

Data collected via Qualtrics was analyzed using SPSS version 26.0 (IBM Corp., Armonk, NY, USA) [42]. Descriptive statistics were computed for participants in each Delphi stage and the entire sample. Continuous variables are summarized using mean (standard deviation) or median (interquartile range), while categorical variables are described using proportions (%) and frequencies. Thematic analysis was employed to analyze responses to the open-ended questions [43]. All responses were first organized by question in a Word document. Suggestions provided under the same open-ended question were then identified, extracted, and compared. Similar suggestions were grouped into preliminary meaning units and further synthesized into actionable revision points based on conceptual similarity. These potential revision points were discussed within the research team and integrated into the program manual when deemed relevant, feasible, and consistent with the program objectives.

#### 2.2.2. Methods for Understanding the Context

Context is a multifaceted concept encompassing cultural, economic, social, and organizational characteristics; geographical settings; and policies [27,44]. This study employed several methods to understand the context, including primary data collection through a Delphi survey (see Section 2.2.1), reviewing published information about Hong Kong (e.g., geographical location, dietary habits) [45] and engaging stakeholders, including depressed adults, in reviewing teaching materials (see Section 2.2.3). Additionally, the Context and Implementation of Complex Interventions (CICI) framework was utilized to provide a comprehensive understanding of the context for the program [46] (see Supplementary Table S2).

#### 2.2.3. Methods for Designing and Refining the Program

The design phase is a creative aspect of program development. The content was developed based on established steps and informed by research on multicomponent lifestyle medicine programs for depression. The format (a group format with 3–10 participants), dose (six 120 min consecutive weekly sessions), and delivery (face-to-face modality, using motivational interviews, the teach-back method, and goal-setting strategies) of the program were based on previous studies [14,16,17,18], health behavior change literature [47,48], the systematic review depicted in Step 1 [25], and discussions with stakeholders and our multi-professional research team (one psychiatrist, one clinical psychologist, two mental health nurses, one TCM nurse, and eight Hong Kong Chinese medicine practitioners).

Subsequently, the research team, three program providers (demographic characteristics shown in Supplementary Table S3), and five Hong Kong Chinese depressed adults (demographic characteristics shown in Supplementary Table S4) evaluated and refined the initial program manual to assess its feasibility, acceptability, and engagement [35]. Depressed patients were voluntarily recruited through convenience sampling from other community-based depression-related projects, based on their availability and willingness to provide feedback on the draft program manual. Depression was identified through self-report, validated screening questionnaires, or physician diagnosis. Depression severity was not formally assessed, as the consultation focused on experiential feedback rather than clinical evaluation. Data were collected using open-ended questions. Example questions included: “what suggestions for improving the six sessions and the whole program?”, and “do you think that the structure and content of each program session is suitable?” The questions focused on the acceptability, clarity, relevance, and feasibility of the program content. Revisions to the program manual were made based on the qualitative feedback collected.

### 2.3. Methods—Step 3: Drawing on Existing Theories and Articulating Program Theory

In this step, we aimed to integrate existing theories into the program theory for the multicomponent TCM lifestyle medicine program for depression, presenting it within a conceptual framework and corresponding logic model.

#### 2.3.1. Methods for Drawing on Existing Theories

Using existing theories helps researchers identify feasible and relevant elements to achieve the program’s intended goals and informs the content and delivery [49]. The research team reviewed various behavior change and psychological theories that support lifestyle-related programs, including the theory of reasoned action, theory of planned behavior, integrated behavior model, health belief model, transtheoretical model (TTM), social cognitive theory (SCT), and socioecological model [47,50,51].

#### 2.3.2. Methods for Articulating Program Theory

Program theory explains how the program under development aims to produce health outcomes [49]. A conceptual framework is intertwined with program theory and serves as the theoretical foundation for program theory [52]. The conceptual framework for our program was created based on theoretical sources (stages of change and several theoretical constructs from the TTM) and empirical sources (literature and books on TCM health preservation and lifestyle medicine, and TCM clinical practice guidelines for depression treatment) (Table 1).

A logic model, which illustrates the pathways from program activities to outcomes over time [35], was adopted to present our program theory, guided by existing development resources and influenced by several logic model templates [53,54], including those applied in complex programs [55]. The quality of the developed logic model was assessed using the logic model review checklist [56] (see Supplementary Table S5).

## 3. Results

The final program reflected the sequential integration of literature evidence, Delphi consensus, and stakeholder feedback. Literature and existing evidence informed the initial intervention components and Delphi questionnaire items; Delphi findings validated, refined, and prioritized the intervention content; stakeholder feedback further improved the feasibility, acceptability, clarity, relevance, and engagement of the program manual.

Overall, the development process generated three key findings. First, the program content was organized around three TCM health preservation themes—*nourishing the heart*, *nourishing according to time*, and *nourishing the Qi*—which were considered theoretically relevant to depression and practically applicable for individuals with depressive symptoms. Second, expert consensus reduced and refined the initial content pool into a more focused set of core intervention items, suggesting that the final program retained components considered most important and appropriate by Chinese medicine practitioners. Third, stakeholder input strengthened the contextual relevance and scientific rationale of the program, supporting its potential feasibility as a structured multicomponent TCM lifestyle medicine intervention for depression.

### 3.1. Results—Step 1: Preparation Phase

#### 3.1.1. Results of Understanding the Public Health Issue and Identifying Resources

The need for a novel multicomponent TCM lifestyle medicine program for depression, an urgent public health issue, was confirmed after our literature review.

#### 3.1.2. Results of Reviewing Published Evidence

According to the updated textbook on health preservation [22], three themes of the program were selected through team discussion and experts’ opinions: *nourishing the heart* (theme 1), *nourishing according to time* (theme 2), and *nourishing the Qi* (theme 3). The selection reflects the TCM dialectical treatment and nursing and is closely related to mood, particularly in relation to depression. Based on the integrated evidence from three resources, an initial seven-point Likert-type scale comprising 61 items for evaluating the program content in the Delphi survey was constructed (40 items for Theme 1, 12 items for Theme 2, 9 items for Theme 3). The systematic review highlighted a lack of evidence-based and theory-informed development in previous programs [25]. Also, the information that the average program duration was 6.5 weeks (2–12 weeks) was reported. These insights provided directions for the development of the multicomponent TCM lifestyle medicine program for depression.

### 3.2. Results—Step 2: Validating Program Components and Content Within the Understanding the Context

#### 3.2.1. Results of Delphi Survey

##### Expert Panel Demographics

Ten Chinese medicine practitioners participated in the three-round Delphi survey from 1 September to 12 November 2022. The response rate was 83.33% (out of 12 experts), and the retention rate was 100%. The demographic characteristics of the 10 Delphi panel members are shown in Table 2.

##### Round 1

The first-round survey took place from 1 September to 9 September 2022 (nine days). A consensus was reached on 42 items after the first-round survey, which included 26 for *nourishing the heart* (e.g., “Introduce the syndrome differentiation and treatment of TCM health preservation and emotion”); 8 for *nourishing according to time* (e.g., “Describe TCM theories and knowledge related to sleep, including sleep stages, the role of sleep, and the assessment of sleep quality”); and 8 for *nourishing the Qi* (e.g., “Describe dietary suggestions related to TCM emotion and depression for *nourishing Qi*”). The median, interquartile range, and percentage agreement values in the Round 1 Delphi survey are presented in Supplementary Table S6.

##### Round 2

The second-round survey commenced on 30 September and ended on 9 October 2022. In this round, 8 items showed agreement of ≥80%. Fourteen items with less than 80% agreement were removed, and the reasons provided by experts are listed in Supplementary Table S7. The median, interquartile range, and percentage agreement values in the Round 2 Delphi survey are presented in Supplementary Table S8.

##### Round 3

The third-round survey was conducted from 4 November to 12 November 2022. “Theoretical knowledge of TCM health preservation” was given the highest priority among the four dimensions of the program (see Supplementary Table S9). Twenty-six items were established as the consented content of the program (Table 3).

#### 3.2.2. Results of Understanding the Context

The context suitability of the initial program content was enhanced through adjustments based on various perspectives (revision examples shown in Supplementary Table S10).

#### 3.2.3. Results of Designing and Refining the Program

Three rounds of refinements regarding the program objectives, content arrangement, and time allocation were conducted among the research team. Five iterative refinements were conducted with three Chinese medicine practitioners, followed by one iteration with depressed patients. Specific examples of changes included adding information such as “not drinking and consuming food one hour before bedtime” and “Adding more examples of seasonal food based on different seasons in the teaching materials of *nourishing according to time.*” (see Supplementary Table S11). The final program consisted of three components: *nourishing the heart* (Component 1), *nourishing according to time* (Component 2), and *nourishing the Qi* (Component 3) (see Table 4 for details). A final program manual was developed (see Supplementary File pages 28–52, Figures S2–S7).

Each module follows the same structure: introduction or warm-up, a pre-session knowledge test, the main session content, a 10 min break, checking, a summary, a question-and-answer session, a post-session knowledge test, and SMART goal setting. Herbal tea tasting and herbal tea packets are provided in the first four modules.

### 3.3. Safety Considerations

Safety procedures will be incorporated to minimize potential harms associated with the program components. Participants with notable suicidal ideation or severe depressive symptoms will be immediately provided with referral information and 24 h emotional support hotlines, with follow-up by a research assistant. For acupressure, participants will be advised to apply gentle pressure, avoid injured or inflamed areas, and stop if discomfort occurs. For *Baduanjin* and other light exercises, participants will be instructed to practice within their physical capacity and discontinue if dizziness, chest discomfort, shortness of breath, pain, or excessive fatigue occurs. For meditation and emotional regulation practices, participants will be advised to pause the activity and seek support if emotional distress intensifies.

### 3.4. Results—Step 3: Drawing on Existing Theories and Articulating Program Theory

#### 3.4.1. Results of Drawing on Existing Theories

The TTM, an integrative behavior change model developed based on multiple theories from various disciplines like SCT and learning theory, was drawn on for our program because its characteristics are suitable for the multicomponent TCM lifestyle medicine program and the research aim [48,51,52,57]. Integrating theories from multiple disciplines can enrich program development [35].

#### 3.4.2. Results of Articulating Program Theory

**Figure 2:**
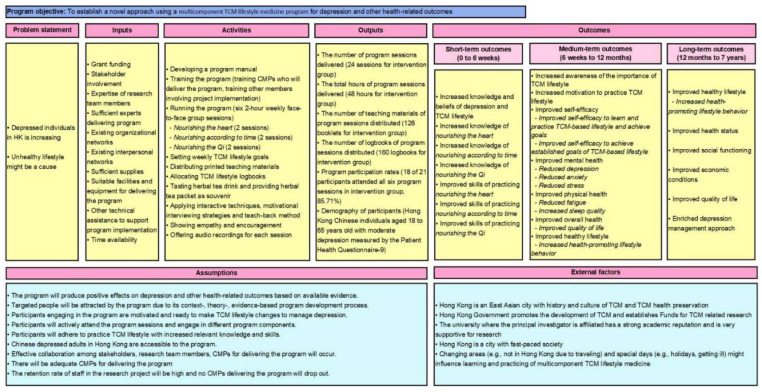
Logic model of multicomponent TCM lifestyle medicine program for depression. Note. CMPs = Chinese Medicine Practitioners; TCM = Traditional Chinese Medicine.

The conceptual framework for the multicomponent TCM lifestyle medicine for depression was developed with nine explanations and corresponding assumptions (see Supplementary Table S19). Guided by the program theory, a logic model was created (Figure 2).

The logical model shows how funding, stakeholder engagement, research team expertise, experts delivering the program, networks, facilities, supplies, equipment, technical support, and time enable program development and implementation. These inputs support manual development, provider and staff training, six weekly group sessions, goal setting, teaching materials, logbooks, herbal tea activities, motivational interviewing, teach-back, empathy, encouragement, and audio recordings. These activities are expected to improve participants’ TCM-related knowledge, beliefs, and practical skills in the short term, which then enhance motivation, self-efficacy, goal achievement, health-promoting behaviors, mental health- as well as physical health- related outcomes in the medium term. Sustained use of TCM lifestyle practices is expected to contribute to long-term improvements in lifestyle, health status, social functioning, quality of life, economic conditions, and depression management.

## 4. Discussion

This study demonstrated the implementation of the updated MRC framework guidelines to develop an evidence-based, theory-informed, implementable, and practicable complex program for depression. Existing multicomponent lifestyle-based interventions, particularly those developed in Western contexts, have shown promising effects in the management of depression through components such as physical activity, dietary modification, smoking cessation, sleep hygiene, and stress management [14,16,17,18,58]. Similarly, TCM-based lifestyle interventions may offer greater cultural relevance for populations familiar with or receptive to TCM concepts by incorporating concepts such as body constitution, dietary regulation, emotional regulation, sleep–wake balance, mind–body practices, and holistic health maintenance [25].

The multisystem coupling approach embedded in the TCM lifestyle intervention, including sleep, stress, and physical activity, is further supported by the systems-level physiological framework of the Network Physiology of Exercise [59,60]. However, existing lifestyle medicine programs, both Western and TCM-based, have rarely provided a sufficiently detailed account of the development process of these complex programs. In particular, limited information has been reported on how intervention components were selected, how the theoretical and practical rationale underpinning program design was established, how Western lifestyle medicine principles or TCM-informed concepts were integrated with empirical evidence, how cultural adaption was conducted, and how feasibility and implementation issues were considered [17,58]. This lack of detailed reporting may lead to research waste [35]. Moreover, substantial heterogeneity has been observed across lifestyle medicine programs for depression, including differences in intervention components, intensity, and duration [17,58]. Such heterogeneity makes it difficult to compare findings across studies and underscores the need for clearer reporting of how intervention components are selected, combined, and prepared for evaluation [17,18]. The present study addresses this methodological gap by explicitly and transparently documenting how a multicomponent lifestyle medicine program was developed through the integration of empirical evidence, theory, clinical expertise, TCM-informed concepts, stakeholder input, expert opinions, and contextual considerations. By applying the updated MRC framework, this study provides a practical example of how a complex health intervention can be systematically developed in the field of complementary and alternative medicine [27,35]. This way, it offers detailed information on the rationale, structure, and development process of the program, which may inform the design of similar interventions in related contexts and support the replication of future randomized controlled trials. Such transparent reporting may enhance reproducibility, reduce research waste [35], and strengthen the credibility of evidence generated in subsequent evaluation studies.

Program theory is the foundation of theory-based evaluation, a form of evaluation that addresses the limitations of traditional evaluation methodologies within the framework of positivist scientific understanding and not only assesses whether a program works but also how a program is expected to generate findings [61]. However, a dearth of studies (particularly in lifestyle medicine for depression) have not formulated or demonstrated a program theory to elucidate how the program’s active components were expected to function and the impacts the context brought [61]. The current study is based on a theoretical source and an empirical source to develop a program theory for the multicomponent TCM lifestyle medicine program for depression, followed by creating a visual logic model. This works as an example of developing program theory and promoting further theory-based evaluation. This theory-based action is rooted in critical realism [35] and is consistent with the philosophical underpinnings of the development and evaluation of the multicomponent TCM lifestyle medicine for depression, demonstrating the rigor of the research design.

Based on the published evidence, findings from the Delphi survey, discussions with stakeholders, and the multi-professional research team, we successfully developed a program manual for the multicomponent TCM lifestyle medicine for depression. The program involves three major themes, each with six 120 min weekly consecutive sessions: *nourishing the heart* (two sessions), *nourishing according to the time* (two sessions), and *nourishing the Qi* (two sessions). The program covered TCM theory and practice to promote healthy sleep, diet, stress management, and self-administered acupressure and TCM exercise. It should be noted that the Delphi method has been widely adopted to obtain expert consensus in TCM [62,63]. In addition, the developed program manual can assist program providers and other research members (e.g., research assistants who will collect data) in understanding, delivering, or replicating the program [35]. It will also function as a basic material for further program training.

Understanding the context while developing the program may reduce the risk of failure in later research stages, such as program evaluation or implementation [35]. Nevertheless, in the realm of lifestyle medicine for mental health, limited attention has been given to the issue of context during the process of developing multicomponent lifestyle medicine programs for depression [14,16,17,18]. In these studies, the main actions taken to understand the context were only simple adjustments of the protocol, which has been developed and validated in different countries influenced by various histories, cultures, economics, and health care systems (protocols utilized in the Healthy Body Healthy Mind Integrative lifestyle program in Australia are an example) [14,16,64]. The main focus of protocol adaptation was diet rather than other areas, such as physical activity, sleep education, and stress management [16,18]. By contrast, our study involved a diverse group of members who provided comprehensive perspectives on the Hong Kong context. Meanwhile, the CICI framework was applied to help us understand and analyze the context throughout the program development. Moreover, revisions based on findings from three approaches (the Delphi survey, a published evidence review, and reviews by program providers and depressed individuals) were completed. Thus, those strategies together will comprehensively enhance the suitability of the program used in Hong Kong, not only in the diet domain, but also in other lifestyle-related domains. That might thereby enhance the likelihood of acceptance, use, and adherence to the practice of the developed novel treatment among individuals with depression in corresponding regions, contributing to better depression management and the improvement of overall well-being.

During the Delphi process, some initially proposed items were ultimately not retained in the final program content. For instance, the item on introducing the “*Ziwuliuzhu*” (midnight–noon ebb–flow) method in the context of sleep hygiene was excluded. “*Ziwuliuzhu*” theory advocates taking a short nap from 11:00 to 13:00. Its theoretical underpinnings and clinical applicability remain contentious within the scholarly community. It also contradicts traditional sleep hygiene education [65]. Similarly, the suggestion to present “refined hobbies” for *nourishing the heart*, such as angling, was removed due to practical limitations in demonstrating these activities in a classroom setting. Moreover, the item on using TCM aromatherapy (e.g., chamomile, lemon, or their mixtures) as a sleep aid was not adopted, as aromatherapy is generally not regarded as a traditional TCM health preservation technique and was therefore considered inconsistent with the program’s theoretical framework. Such refinement will help make our program better fit the Hong Kong context and, in turn, enhance its applicability. Our later qualitative study in collecting feedback from participants also revealed that the course contents are feasible and have helped them to make changes in lifestyle [66].

Although the first phase (intervention development phase) of the updated MRC framework has been primarily reached in our program development, the iterative nature of program development should be acknowledged. Significant problems identified in the later phases may require researchers to return to the development phase and further refine the program [35]. Such iterations may occur several times, and the intensive development process may stop when only limited refinements are recommended by program deliverers, participants, or researchers observing the program’s implementation [35]. In addition, future implementation of the program may face challenges related to scalability, provider training, and resource requirements. In particular, standardized training for program facilitators, the quality control of TCM-related components, and the costs associated with staff time, materials, and session delivery should be considered before wider dissemination. When the second phase of program evaluation is completed, the Template for Intervention Description and Replication Checklist will be used to report the program [67]. This, together with the current paper and program manual, will facilitate policymakers, service providers, and developers external to our research team in evaluating, adapting, or implementing the program.

### Strengths and Limitations

This study has several strengths. First, we systematically developed a complex TCM-based health program for depression by integrating multiple evidence sources and relevant theoretical frameworks, following a structured development process. Second, we engaged a range of stakeholders—including researchers from different disciplines, clinicians, program providers, and patients—throughout the development process to enhance the relevance, acceptability, and feasibility of the intervention. Third, we explicitly considered contextual factors in Hong Kong, such as local health care practices and patient preferences, which may facilitate subsequent implementation in real-world settings. These features may provide a useful reference for the future development of complex TCM health interventions.

Nonetheless, the present study has some limitations. First, the Delphi panel was relatively homogeneous in disciplinary background, consisting of Hong Kong Chinese medicine practitioners. This may have limited the extent of input regarding mental health assessment, symptom monitoring, and risk management. To mitigate this limitation, the psychiatrist and clinical psychologist on the research team were consulted during program development, and referral procedures were established for program participants with notable suicidal ideation or severe depression.

Second, the sample size in the qualitative refinement phases was limited, which may have constrained the diversity of feedback obtained, as only three Chinese medicine practitioners and five adults with depression participated. Those depressed participants were relatively young and highly educated. In addition, as participation was voluntary, depressed participants may have had greater interest in or openness toward TCM-based interventions than the broader target population. Therefore, their views may not fully represent those of individuals with lower educational levels, limited familiarity with TCM, or lower TCM health literacy. Future studies involving a broader stakeholder panel as well as larger and more diverse samples are required to further collect feedback to refine the program content.

Third, as this study focused on program development rather than empirical evaluation, the feasibility, acceptability, engagement, and effectiveness of the program require formal testing in future pilot or feasibility studies.

Lastly, given its development within the Hong Kong Chinese context and its incorporation of culturally relevant lifestyle medicine and Traditional Chinese Medicine elements, the program’s transferability to non-Chinese populations or other sociocultural contexts may not be plausible.

## 5. Conclusions

This article reports the development of a complex, multicomponent TCM lifestyle medicine program intended to relieve depressive symptoms, with attention to the Hong Kong context and TCM theory. The program was developed through a structured process that incorporated multiple sources of evidence and stakeholder input. Future pilot studies and process evaluations are needed to examine its feasibility, acceptability, and preliminary efficacy.

## Figures and Tables

**Table 1:** Sources forming the conceptual framework for multicomponent Traditional Chinese Medicine lifestyle medicine program for depression.

Source	Dimension		
Theoretical source	1. Stages of change (precontemplation, contemplation, preparation, action, and maintenance)	2. Several theoretical constructs from the Transtheoretical Model (self-efficacy, consciousness raising, self-revaluation, helping relationships, reinforcement management, counter conditioning, social liberation, and stimulus control)	
Empirical source	1. A review of Traditional Chinese Medicine health preservation literature and books	2. Lifestyle medicine literature and books for relevant topic	3. Traditional Chinese Medicine clinical practice guidelines of depression treatment

**Table 2:** Demographic characteristics of the Delphi panel members (*N* = 10).

Characteristic	Number	Percentage (%)
Age in years, mean (range; SD)	41 (32 to 52; 5.66)	/
Gender		
Female	6	60
Male	4	40
Education level		
Doctorate degree	8	80
Master’s degree	2	20
Years of working as a CMP ^1^ in Hong Kong		
6–10	3	30
11–15	4	40
16–20	3	30
Working units of local CMP ^1^ s		
Private clinics	3	30
University-affiliated clinics	6	60
Public clinics	1	10

^1^ CMP = Chinese medicine practitioner; SD = standard deviation; / = not applicable.

**Table 3:** The mean and SD values of the importance ratings for the multicomponent TCM lifestyle medicine program in the Round 3 Delphi survey.

Items	Mean (SD ^1^)	Rank
**Section 1: The curriculum setting of nourishing the heart**		
**Introduce the etiology and pathogenesis of TCM health preservation and emotion (depression)**	**6.3 (0.67)**	**1**
**Define mental health care in nourishing the heart**	**6.3 (1.25)**	**2**
**Provide simple, syndrome differentiation-based diet suggestions for depression in class**	**6.3 (0.67)**	**3**
**Introduce the definition of TCM ^2^ health preservation**	**6.1 (1.29)**	**4**
**Introduce the theoretical foundation of TCM ^2^ health preservation**	**6.1 (0.74)**	**5**
**Introduce the therapeutic strategies of TCM ^2^ health preservation and emotion, including nourishing the heart, nourishing the Qi, and nourishing according to time**	**6.1 (0.99)**	**6**
**Practice *Baduanjin*, a method of qi regulation, in class with the instructor**	**6.0 (0.94)**	**7**
**Define refined hobbies in nourishing the heart**	**6.0 (0.82)**	**8**
**Describe the specific method of mental health care in nourishing the heart: Temperance method**	**5.9 (0.74)**	**9**
**Describe the specific method of mental health care in nourishing the heart: Catharsis method**	**5.9 (1.29)**	**10**
**Describe the specific method of mental health care in nourishing the heart: Abstinence and essence conservation, clear heart, and calm the mind**	**5.9 (0.74)**	**11**
**Describe the specific method of mental health care in nourishing the heart: Meditation**	**5.9 (0.57)**	**12**
**Practice meditation, a method of mental health care in class**	**5.9 (0.74)**	**13**
**Describe the specific method of mental health care in nourishing the heart: Qi regulation**	**5.8 (0.63)**	**14**
Describe the specific method of mental health care in nourishing the heart: Spiritual cultivation in the four seasons	5.7 (1.06)	15
Describe the definition and function of social interaction in nourishing the heart	5.7 (1.16)	16
Introduce the syndrome differentiation and treatment of TCM ^2^ health preservation and emotion	5.6 (0.70)	17
Describe the specific method of mental health care in nourishing the heart: Distraction	5.6 (1.07)	18
Describe the specific method of mental health care in nourishing the heart: Enlightened method	5.6 (1.07)	19
Describe the principles and measures of establishing a healthy communicative environment in social interaction for nourishing the heart	5.6 (1.17)	20
Describe a refined hobby for nourishing the heart: Travel	5.5 (1.18)	21
Describe a refined hobby for nourishing the heart: Music therapy	5.4 (1.26)	22
Try a refined hobby by appreciating a beautiful painting and/or calligraphy that could nourish the heart	5.2 (1.14)	23
Describe a refined hobby for nourishing the heart: Appreciation of flowers and birds	5.2 (1.23)	24
Describe a refined hobby for nourishing the heart: Aromatherapy	5.1 (1.66)	25
Describe a refined hobby in nourishing the heart: Reading beautiful articles	5.0 (0.82)	26
Describe a refined hobby for nourishing the heart: Tasting tea	4.9 (1.66)	27
Describe a refined hobby for nourishing the heart: Playing chess	4.7 (1.06)	28
**Section 2: The curriculum setting of *nourishing according to time***		
**Describe the concepts and principles of health-preserving daily schedule, which includes conforming to the laws of nature, complying with the patterns of human body, and maintaining the moderation of works and rests**	**6.5 (0.71)**	**1**
**Learn and practice acupoints that can improve sleep quality and relieve depression in class, including Baihui (DU20), Shenmen (HT7), Neiguan (PC6), and Yintang (EX—HN3)]**	**6.4 (0.97)**	**2**
**Describe 3–4 acupoints that can improve sleep quality, relieve depression, and be easy manipulated [Baihui (DU20), Shenmen (HT7), Neiguan (PC6), and Yintang (EX—HN3)]**	**6.3 (0.95)**	**3**
**Describe the principle of eating according to time and season, and introduce seasonal food**	**6.3 (0.67)**	**4**
**Describe sleep regulation methods, including “regulation before bedtime,” “regulation at bedtime,” and “ten contraindications regarding sleep”**	**6.1 (0.74)**	**5**
**Describe self-regulating sleep aids (meditation, self-hypnosis to fall asleep, tranquility)**	**6.0 (0.67)**	**6**
Describe a sleep aid, diet for better sleep; for example, taking a small amount of food for better sleep, such as lily, longan, and jujube (appropriate type of syndrome and body constitution should be considered)	5.9 (0.74)	7
Practice nourishing according to time through tasting shen-calming foods and drinks (suitable food should be provided based type of syndrome and body constitution)	5.9 (0.74)	8
Describe a sleep aid, shen-calming music	5.7 (1.06)	9
Close the eyes and listen to calming music to experience nourishing according to time in class (e.g., five-tone music)	5.7 (1.34)	10
Describe the TCM theories and knowledge in sleep, including sleep stages, the role of sleep, and the judgment of sleep quality, etc.	5.3 (1.34)	11
**Section 3: The curriculum setting of *nourishing the Qi***		
**Define and describe the function of nourishing the *Qi***	**6.2 (1.23)**	**1**
**Describe the relationship between “nourishment *Qi*” and “depression” in TCM (“adjustment of body,” “regulation of breathing,” and “regulation of mental activities”)**	**6.2 (0.79)**	**2**
**Describe diet suggestions related to TCM ^2^ emotion and depression and nourishing**	**6.2 (0.63)**	**3**
**Describe *Baduanjin*, a method for nourishing the *Qi*, including overview, function, and practical method**	**6.1 (0.74)**	**4**
**Experience nourishing the *Qi* by learning and practicing *Baduanjin* in class (twice)**	**6.0 (1.15)**	**5**
**Describe the relationship between Qigong and depression and the related function of Qigong on depression**	**5.9 (0.74)**	**6**
Experience nourishing the *Qi* by learning and practicing six-character formula in class (twice)	5.8 (1.14)	7
Describe the philosophical foundation of Qigong in nourishing the *Qi*	5.7 (0.82)	8
Describe six-character formula, a method for nourishing the *Qi*, including overview, function, and practical method	5.7 (1.06)	9
Describe basic techniques of simple qigong, a method for nourishing the *Qi*, including overview, function, and practical method	5.6 (0.84)	10
Experience nourishing the *Qi* by learning and practicing basic techniques of simple Qigong in class (twice)	5.3 (1.34)	11

^1^ SD = standard deviation; ^2^ TCM = Traditional Chinese Medicine. The twenty-six items that had been established as the consented content of the program are bold.

**Table 4:** Summary of final program components.

Program Component	Component Name	Session Content	Session Structure
Component 1	Nourishing the heart 1	Definition and function of health preservation and TCM health preservation, theoretical foundation of TCM health preservation, etiology, and pathogenesis as well as therapeutic strategies of TCM health preservation and emotion (depression), refined hobbies in *nourishing the heart* 1, practicing *Baduanjin*, providing syndrome differentiation-based diet suggestions for depression (1) and tasting a herbal tea drink (tangerine peel tea)	Introduction/warm-up and recap; pre-session knowledge test; main session content; break; checking, summary, Q&A; post-session knowledge test; SMART goal setting
Component 1	Nourishing the heart	Definition of mental health care in *nourishing the heart*, the specific method of mental health care in *nourishing the heart* (temperance method, catharsis method, *Qi* regulation, and meditation as well as abstinence and essence conservation, clear heart, and calm the mind), practicing meditation, providing syndrome differentiation-based diet suggestions for depression, and (2) tasting a herbal tea drink (rose drink)	Same structure as above
Component 2	Nourishing according to time 1	Concept and manifestation of *nourishing according to time*, concepts and principles of health-preserving daily schedule, “regulation before bedtime” (one sleep regulation method), introducing one acupoint (*Baihui*) that can improve sleep quality and relieve depression, learning and practicing one acupoint (*Baihui*) that can improve sleep quality and relieve depression, providing syndrome differentiation-based diet suggestions for depression (3) (diet for better sleep), and tasting a herbal tea drink (longan tea) (diet for better sleep)	Same structure as above
Component 2	Nourishing according to time 2	Introduction of “regulation at bedtime”, “ten contraindications regarding sleep”, two sleep regulation methods; principle of eating according to time and season, and introducing seasonal food; self-regulating sleep aids; introducing acupoints (*Yintang*, *Neiguan*, *Shenmen*) that can improve sleep quality and relieve depression, learning and practicing one acupoint (*Yintang*, *Neiguan*, *Shenmen*) that can improve sleep quality and relieve depression; providing syndrome differentiation-based diet suggestions for depression (4) and tasting a herbal tea (red date tea) for alleviating depression (4) in class (diet for better sleep)	Same structure as above
Component 3	Nourishing the Qi 1	Definition and function of *nourishing the Qi*, the relationship between “nourishment *Qi*” and “depression” in TCM, the relationship between Qigong and depression and the function of Qigong in depression, Experience nourishing *Qi* by learning and practicing *Baduanjin* in class, and introduce a diet related to TCM emotion and depression and nourishing the *Qi* 1	Same structure as above
Component 3	Nourishing the Qi 2	Diet suggestions related to TCM emotion and depression and nourishing the *Qi* 2; overview, function, and practical method of *Baduanjin*; experience nourishing the *Qi* by learning and practicing *Baduanjin* in class; and overall review of content of sessions of TCM health preservation program for depression	Same structure as above

Note: Numbers in the Program Component column indicate the program component number, with three components numbered 1, 2, and 3. Numbers in the Component Name column indicate the part number within each component, as each component consists of two parts, numbered 1 and 2.

## Data Availability

The data presented in this study are available on request from the corresponding author due to privacy restriction.

## Supplementary Materials

The following supporting information can be downloaded at: https://www.mdpi.com/article/10.3390/healthcare14121631/s1, Figure S1: The flowchart of the Delphi survey design; Figure S2: *Nourishing the heart 1*; Figure S3: *Nourishing the heart 2*; Figure S4: *Nourishing according to the time 1*; Figure S5: *Nourishing according to the time 2*; Figure S6: *Nourishing the Qi 1*; Figure S7: *Nourishing the Qi 2*; Table S1: CREDES Checklist; Table S2: The context and setting dimensions of the multicomponent traditional Chinese medicine lifestyle medicine for depression guided by the context and implementation of complex intervention framework; Table S3: Demographic characteristics of CMPs delivering the multicomponent TCM lifestyle medicine program for depression; Table S4: Demographic characteristics of five people with depression contributing to the program refinement; Table S5: Logic model review checklist for the multicomponent traditional Chinese medicine lifestyle medicine for depression; Table S6: The median, IQR, and percentage agreement values in round 1 Delphi survey; Table S7: Fourteen items removed in Round 2 and related reasons; Table S8: The median, IQR, and percentage agreement values in round 2 Delphi survey; Table S9: Rank of dimensions for development of multiconponet traditional Chinese medicine lifestyle medicine program for depression; Table S10: Examples of content revised or added to fit the Hong Kong context; Table S11: Examples of comments during the iterations of refinement and relevant modifications for multicomponent TCM lifestyle medicine program for depression; Table S12: The whole structure of multicomponent TCM lifestyle medicine program for depression; Table S13: The structure and content of nourishing the heart 1; Table S14: The structure and content of nourishing the heart 2; Table S15: The structure and content of nourishing according to time 1; Table S16: The structure and content of nourishing according to time 2; Table S17: The structure and content of nourishing the Qi 1; Table S18: The structure and content of nourishing the Qi 2; Table S19: Explanations and corresponding assumptions of conceptual framework for the multicomponent traditional Chinese medicine lifestyle medicine program.
