# Supplementary material for: Development of a Traditional Chinese Medicine Lifestyle Medicine Program for Depression: A Multi-Method Study
Source: Healthcare (Basel). 2026 Jun 9;14(12):1631. doi: 10.3390/healthcare14121631 (PMC13299938; doi:10.3390/healthcare14121631)
Supplement: Supplementary file 1 [file healthcare-14-01631-s001.zip › healthcare-4213320-supplementary.pdf]

Supplementary Materials

Figure S1. The flowchart of the Delphi survey design.

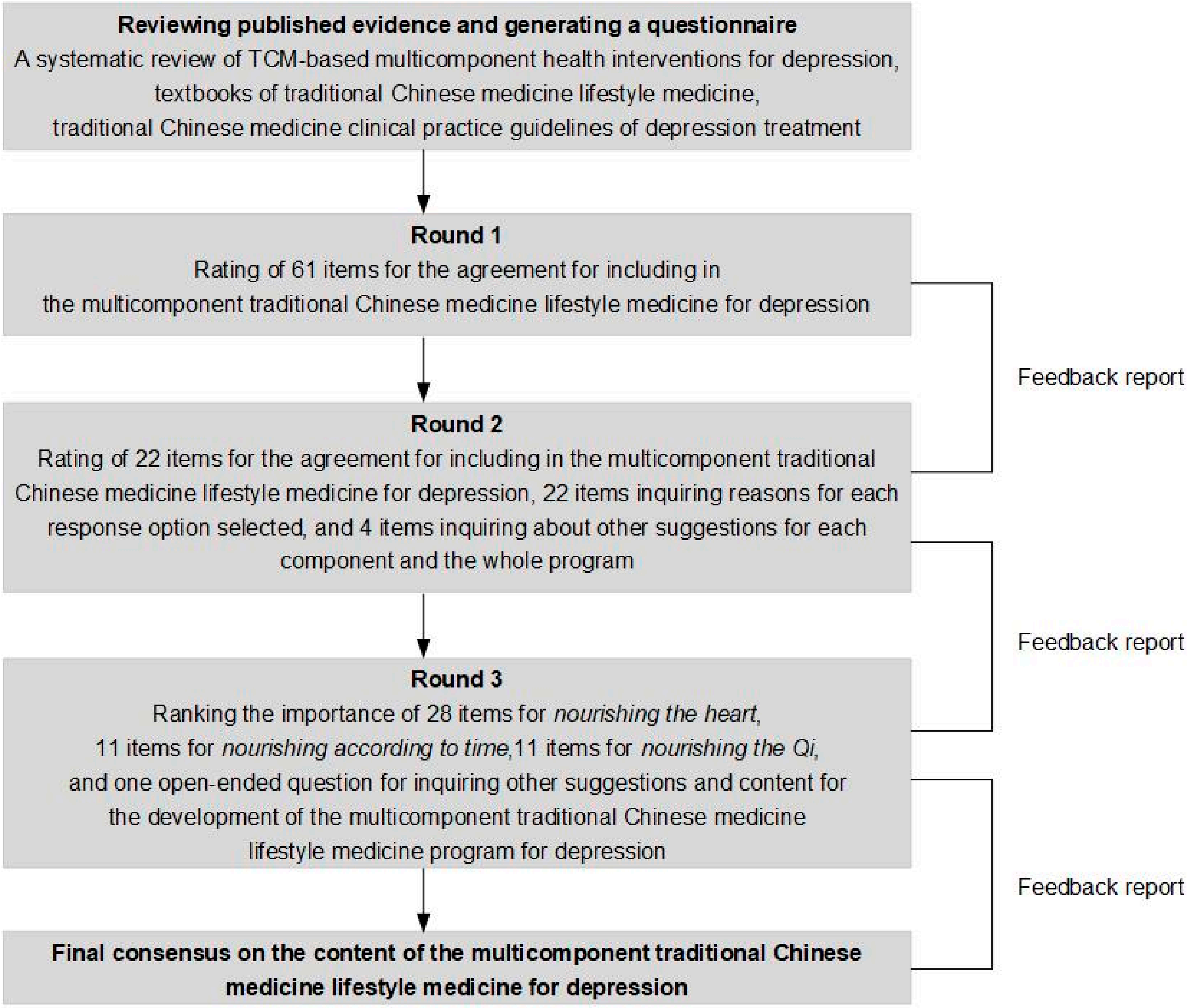

Note. TCM = traditional Chinese medicine.

Table S1. CREDES Checklist.

Guidance on Conducting and Reporting Delphi Studies (CREDES) Checklist [1]	
**Rationale for Delphi Technique**	
Justification. The choice of the Delphi technique as a method of systematically collating expert consultation and building consensus needs to be well justified.	9
**Planning and Design**	
Planning and process. The Delphi technique is a flexible method and can be adjusted to the respective research aims and purposes. Any modifications should be justified by a rationale and be applied systematically and rigorously	9-11
Definition of consensus. Unless not reasonable due to the explorative nature of the study, an a priori criterion for consensus should be defined. This includes a clear and transparent guide for action on (a) how to proceed with certain items or topics in the next survey round, (b) the required threshold to terminate the Delphi process and (c) procedures to be followed when consensus is (not) reached after one or more iterations	9-11
**Study Conduct**	
Informational input. All material provided to the expert panel at the outset of the project and throughout the Delphi process should be carefully reviewed and piloted in advance in order to examine the effect on experts' judgements and to prevent bias.	9-11
Prevention of bias. Researchers need to take measures to avoid directly or indirectly influencing the experts' judgements. If one or more members of the research team have a conflict of interest, entrusting an independent researcher with the main coordination of the Delphi study is advisable.	9-11
Interpretation and processing of results. Consensus does not necessarily imply the 'correct' answer or judgement; (non)consensus and stable disagreement provide informative insights and highlight differences in perspectives concerning the topic in question.	9-11
External validation. It is recommended to have the final draft of the resulting guidance reviewed and approved by an external board or authority before publication and dissemination.	Done
**Reporting**	
Purpose and rationale. The purpose of the study should be clearly defined and demonstrate the appropriateness of the use of the Delphi technique as a method to achieve the research aim. A rationale for the choice of the Delphi technique as the most suitable method needs to be provided.	13-18
Expert panel. Criteria for the selection of experts and transparent information on recruitment of the expert panel	13-18
Description of the methods. The methods employed need to be comprehensible; this includes information on preparatory steps, piloting of material and survey instruments, design of the survey instrument(s), the number and design of survey rounds, methods of data analysis, processing and synthesis of experts' responses to inform the subsequent survey round and methodological decisions taken by the research team throughout the process.	13-18
Procedure. Flow chart to illustrate the stages of the Delphi process, including a preparatory phase, the actual 'Delphi rounds', interim steps of data processing and analysis, and concluding steps.	14-15
Definition and attainment of consensus. It needs to be comprehensible to the reader how consensus was achieved throughout the process, including strategies to deal with non-consensus.	14-15
Results. Reporting of results for each round separately is highly advisable in order to make the evolving of consensus over the rounds transparent. This includes figures showing the average group response, changes between rounds, as well as any modifications of the survey instrument such as deletion, addition or modification of survey items based on previous rounds	13-21
Discussion of limitations. Reporting should include a critical reflection of potential limitations and their impact of the resulting guidance	31-32
Adequacy of conclusions. The conclusions should adequately reflect the outcomes of the Delphi study with a view to the scope and applicability of the resulting practice guidance	32
Publication and dissemination. The resulting guidance should be clearly identifiable from the publication, including recommendations for transfer into practice and implementation. A dissemination plan should include endorsement of the guidance by professional associations and health care authorities to facilitate implementation	Done

1. Jünger S, Payne SA, Brine J, Radbruch L, Brearley SG. Guidance on Conducting and REporting DElphi Studies (CREDES) in palliative care: Recommendations based on a methodological systematic review. Palliat Med. 2017;31: 684–706. doi:10.1177/0269216317690685

Table S2. The context and setting dimensions of the multicomponent traditional Chinese medicine lifestyle medicine for depression guided by the context and implementation of complex intervention framework.

Context			Interactions
**Socio-economic context**	This domain incorporates the community's economic resources and a population's access to these resources (Damschroder et al., 2019; Victora et al., 2005). It also reveals the association between an economy and its society (Damschroder et al., 2019; Victora et al., 2005).	**Macro (National and International level)**<br>China, a country located in East Asia, is the second world most populous country, ranking as the United States (25464.48 billion), China (18100.04 billion, occupying 7.97% of the world economy), and Japan (4233.5 billion U.S dollars), according to the GDP in 2022 (O'Neil, 2023). In 2021, the GDP value of China was 17.82 trillion U.S dollars (Macrotrends, 2024). That year (2021), the total value of C industry has reached to 700 billion, showing a big increase compared to 515.6 billion in 2012 (Xinhua Finance, 2022). Similar increase could be reflected from the output value of TCM, showing as the output value of TCM amounted to ren min bi 317.2 billion in 2010 and the estimated total TCM market in China increasing to €96.2 billion in 2025 (Xu & Xia, 2019). The net produced and profits of TCM production are much larger than the average for the medical industry of the country (Xu & Xia, 2019).<br>**Meso (City level)**<br>• **GDP of Hong Kong in 2022:** in 2022, the GDP at current market prices of Hong Kong was 2,818,046 Hong Kong dollar million, the GDP in chained dollars was 2,767,934 Hong Kong dollar million (Census and Statistics Department, 2023b).<br>• **Government subsidizing TCM service:** Since March 2020, the daily operating expenses of CMCTRs and out-patient services at these clinics are subsidized by the government (The Hong Kong Special Administrative Region of the People's Republic of China, 2020). The annual provision for the operation of CMCTRs more than doubled from 94.5 million Hong Kong dollars to 230 million Hong Kong dollars in 2021 to 2022 (Health Services, 2022).<br>• **An international financial centre:** supported by the Hong Kong Government to enhance its status as an international financial centre, which is highly open and internationalised market (International Financial Centre, 2021).<br>• **TCM retail sales value:** In 2022, the TCM retail sales value of Chinese herbs and drugs in Hong Kong added up to approximately 4.4 billion Hong Kong dollars (Statista, 2023).<br>• **500 million Hong Kong dollars fund for CM development in Hong Kong:** As reported in the "policy Adress" of 2018, a special fund within 500 million Hong Kong dollars has been established to promote the development of TCM (Chief Executive, 2018). It is coordinated by the CM Division under the Food and Health Bureau, supporting applied research, development of specialized CM, promoting knowledge exchange, cross-market collaboration, and assisting local CM traders in the production and registration of Chinese patent medicines (Chief Executive, 2018). The special fund will commence operation in the first half	socio-cultural context, ethical context, legal context, and political context

		of next year" (Chief Executive, 2018).<br>**<u>Micro (Individual level and household level)</u>**<br>• **Income of Hong Kong residents:** in 2022, the per capita GDP in chained dollars was 376,790 Hong Kong dollars (Census and Statistics Department, 2023b). During May to June 2023, the median monthly wage of an individual is 19,100 Hong Kong dollars (Census and Statistics Department, 2023c).<br>• **Unemployment rate:** the unemployment rate in May to July 2023 and June to August 2023 both were 2.8% (Census and Statistics Department, 2023d).<br>• **Price of attending TCM clinics:** The fee for each consultation is set at 120 Hong Kong dollars (Hospital Authority, 2022).	
**Socio-cultural context**	This domain includes implicit and explicit behaviour patterns, incorporating embodiment in artefacts and symbols; the essential key of culture is composed of historically derived and chosen values and ideas that are shared among group member (Sabatier, 2007).	**<u>Macro (National and international level)</u>**<br>**TCM as an integral part of mainstream medicine in China and has spread overseas:** TCM is the treasure of ancient Chinese science and the key to the treasure house of Chinese civilization. TCM was originated in the Yin and Shang dynasties in China 3000 years ago (Ma et al., 2021). Up to present, TCM has spread to 196 countries and regions and gained global recognition for its unique strengths in preventing disease, controlling disease, and rehabilitation (Xinhua, 2023). To provide TCM medical aid overseas, China has sent medical teams which involved TCM professionals in almost every team to more than 70 countries in Africa, Asia, and Latin America (The State Council Information Office, 2016).<br>**<u>Meso (City level)</u>**<br>• **TCM as part of Hong Kong's cultural heritage:** Hong Kong TCM is a branch of Lingan or Cantonese TCM, which shows several unique practices in comparison to other branches of TCM (Lo, 2020). Herbal tea ("cooling tea" or "leung cha") is just one example, which is a decoction of Chinese medicinal herbs made based on TCM theories (Intangible cultural heritage office, 2023). Herbal tea in Hong Kong has more than one hundred years and are representative of traditional culture and folk knowledge (Intangible cultural heritage office, 2023). In 2006, herbal tea was inscribed onto the Hong Kong Intangible Cultural Heritage Database and the inventory code was 4.1.1. (Intangible cultural heritage office, 2023).<br>• **TCM education and CMPs cultivation in Hong Kong:**<br><u>TCM education before and after the second World War</u>**:** Before the second World war, there were several schools established under different names for CM education in Hong Kong (Editorial Committee of The Mirror Elucidating the way of Medicine, 2022). These included Dr. Ho Pui Yu's Ciu Sin Chinese Medical School, Dr. Chan Pak Tam's Pak Tam Chinese Medical Specialist School, Dr. Wong To's Bao Yuan Chinese Medical Specialist School, Dr. Ng Yiu Yin's Hong Kong Guangdong CM School, and Dr. Tsang Tin Chi's Scientific Acupuncture Medical College (Editorial Committee of The Mirror Elucidating the way of	socio-economic context, ethical context, legal context, political context, and geographical context

	It not only means the conditions where people are born, grow, work, live, and age but also refers to the social roles an individual takes on as a citizen, community member or a family member and the relationships inherent to these roles. Constructs such as beliefs, knowledge, institutions, customs, conceptions and any other abilities and habits obtained by a group are belonged to this domain (Lysdahl	Medicine, 2022). After the war, schools emerged like mushrooms after the rain. Both guild and privately-run schools appeared, and they were larger in terms of structure and quantity compared to before the war (Editorial Committee of The Mirror Elucidating the way of Medicine, 2022).<br><u>TCM education since 1998</u>**:** Since 1998, the Hong Kong Baptist University has offered the full-time bachelor of CM and Bachelor of Science (Hons) in biomedical science double degree program funded by University Grants Committee (Bian, 2017). Thereafter, another two universities, the Chinese University of Hong Kong, and the University of Hong Kong, started to offer full-time CM programs (Bian, 2017). The three local universities provided a range of CM graduates (Bian, 2017).<br><u>CMPs cultivation</u>: At end-February 2022, 3,177 applications had been approved for CMPs and CM drug practitioners to attend training programmes (CM Personal Training Funding Scheme) (Health Services, 2022). Applications for "Certification of registration of proprietary Chinese medicine" registration of some 600 proprietary CM had also been provided with subsidy to engage professional consultants and/or conduct the necessary testing to meet the registration requirements (Health Services, 2022).<br>• **The number of CMPs in Hong Kong:** CMPs in Hong Kong are composed of registered CMPs, CMPs with limited registration, and listed CMPs. The number of CMPs in 2015, 2017, and 2019 was 9,787, 10,086, and 10,173, respectively (Health Services, 2022). As at end-February 2022, there were 10,557 CMPs (also known as 14.3 CMPs per 10,000 population) in Hong Kong (Health Services, 2022). The number of CMPs are adequate supply in the short-to-medium term (Health Services, 2022).<br>**<u>Micro (Individual and household level)</u>**<br>• **TCM as a preference by Hong Kong population: Ancient Hong Kong residents:** Ancient Hong Kong residents have already experimented with TCM. Due to the material characteristics, it is difficult to preserve ancient medical relics related to the local area (Editorial Committee of The Mirror Elucidating the way of Medicine, 2022) The only known ancient book directly related to medicine is the Prescription Stele of Cheung Chau (Editorial Committee of The Mirror Elucidating the way of Medicine, 2022). It contains only the name of Yuan Cong Tea and many numerical markings for dosage. Experts speculate that it was a prescription for treating children's diarrhoea (Editorial Committee of The Mirror Elucidating the way of Medicine, 2022).<br>• **Increase demand for CM services:** that increase could be reflected from two aspects. On the one hand, the number of attendances at the 18 CMCTRs reached 1.28 million in 2021, increase by 16% compared to that in 2015 (Health Services, 2022). On the other hand, that rise could be reflected in the rising spending on CM covered by Elderly Health Care Vouchers since 2015 to 2021 (Health Services, 2022).	

	et al., 2016).		
**Ethical context**	The ethical domain indicates morality reflections, which incorporates standards of conduct, beliefs, and principles that guide the behaviour of institutions and individuals (European Network for Health Technology Assessment, 2007)	<u>**Meso level**</u><br>• **Equity to medical services:** The equity to medical services could be demonstrated from three aspects. First, recipients of comprehensive social security assistance/recipients of old age living allowance aged 75 or over will be exempted from charges (Hospital Authority, 2022). Second, 18 CMCTRs were distributed in Hong Kong, showing as one in each district (Health Services, 2022). Specifically, four in Hong Kong Island (Eastern district, Southern district, Wan Chai, Central & Western), five in Kowloon (Kowloon city, Kwun Tong, Sham Shui Po, Wong TaI Sin, Yau Tsim Mong), and nine in NEW territories (Islands, Kwai Tsing, North, Sai Kung, Sha Tin, Tai Po, Tsuen Wan, Tuen Mum, Yuen Long) (Health Services, 2022; Hospital Authority, 2024). Selected districts are those with percentage changes in attendance between 2015 and 2021 that are higher than the average percentage change of all districts (Health Services, 2022). Third, as reported in the "Policy Address" of 2022, additional 200,000 CM outpatient service quotas will be provided (Chief Executive, 2022). Thus, related annual outpatient service quotas will increase from 600,000 to 800,000 (Chief Executive, 2022).<br>• **Inequity to CMPs:** CMPs in Hong Kong mainly work at private CM clinics, and semi-public Chinese services which consisted of non-governmental organizations, Tripartite CM Clinics cum Training and Research Centres, university CM clinics, and others like CM volunteers (Bian, 2017). Large gap between actual salary and expectation, as well as the slow progress in developing a CM speciality structure and an associated training and qualification accreditation mechanism.	Legal context, political context, and socio-economic context
**Legal context**	The legal domain involves with the regulations and rules that have been established to protect a population 's societal interests	<u>**Macro level (National and international level)**</u><br>• **Policies, rules and regulations issued to promote standard TCM management:**<br><u>International:</u> China has facilitated the founding of the ISO/TC429 TCM in the ISO to ensure TCM's safe, efficient, and targeted implementation (The State Council Information Office, 2016).<br><u>National:</u> TCM development has become an integral part of China's implementation of the "Belt and Road" strategy (Xu & Xia, 2019). The Chinese communist Party's 18th National Congress and the Fifth Plenary Session of the 18th Chinese communist Party Central Committee both reiterated the necessity to pay equal attention to the development of TCM and Western medicine (Xu & Xia, 2019). The Outline of the Strategic Plan on the development of TCM (2016-2030) was issued in 2016, making TCM	Political context and socio-cultural context

	and rights (European Network for Health Technology Assessment, 2007).	development a national strategy (The State Council Information Office, 2016).<br>**<u>Meso (city level)</u>**<br>• **The establishment of the CM Ordinance:** In 1999, the CM Ordinance (Cap. 549 of the Laws of Hong Kong) was passed by the Legislative Council and Hong Kong and the CM Council of Hong Kong was officially established (Editorial Committee of The Mirror Elucidating the way of Medicine, 2022) (Bian, 2017).<br>• **Established of CMCTRs:** In 2013, 18 CMCTRs have been established and operated with the subsidization from the government (Health Services, 2022).<br>• **The ICWM Pilot Programme**: In 2014, the Hospital Authority launched the ICWM Pilot Programme to provide ICWM treatment services for in-patients of three chosen disease areas, including stroke care, musculoskeletal pain management and cancer palliative care (Health Services, 2022). The coverage of the ICWM Pilot Programme has gradually expanded from three hospitals in 2014 to the present eight, with 2,866 patients having enrolled in the programme as at end-2021 (Health Services, 2022).<br>• **Constructing the first CM hospital**: the first CM hospital, which will start to provide services by stages from mid-2025, will mainly provide CM services with ICWM services being offered for particular patient types or disease where patients can benefit from both genres of treatment (Health Services, 2022).<br>• **Establish position of "CM Development Commissioner":** in "Policy Address" of 2022, this position established aims to further promote TCM development (Chief Executive, 2022).	
**Political context**	The political domain targets at distributing assets, power, and interests within a population, and the scope of organisations involved, related interests and the	**Macro (National and International level)**<br>• **International**: the WHO has incorporated traditional medicine, presented by TCM, in the ICD-11 on 25 May 2019. Besides, the WHO traditional medicine strategy: 2014-2023 (WHO, 2013) which will extend until 2025 has been conducted (WHO, 2023c).<br>• **National**: Political department: In 1986, the State Council established a relatively independent administrative department for TCM (The State Council Information Office, 2016).<br><u>Laws and regulations</u>: In 2003, the State Council has promulgated and implemented the "Regulation of the People's Republic of China on TCM"; In 2009, the State Council has promulgated and implemented the 'Opinions on Supporting and promoting the Development of TCM'; and in 2016, TCM has paid more attention as an national strategy, reflecting from the Outline of the Strategic Plan on the development of TCM (2016-2030) issued by the State Council (The State Council Information Office, 2016).<br><u>TCM medical and educational resources</u>: in 2021, the number of TCM medical and health institutions reached 77,336 and the same year, the number of higher education institutions for TCM reached 44, higher education institutions for western medicine	Legal context, socio-economic context, socio-cultural context, ethical context and geographic

	informal and formal rules that regulate interactions between them. The domain also consists of the health care system and the securing of its accessibility.	that provide TCM courses reached 152, higher education institutions fields that provide TCM courses reached 259 in China (The State Administration of Traditional Chinese Medicine, 2021).<br>**Meso (City level)**<br>• **Political context during different development stage of TCM in Hong Kong:**<br>The development of TCM in the early days of Hong Kong's port opening (1840-1900): in 1872, Tung Wah Hospital, the first hospital in Hong Kong, was found to provide free CM service for patients ((Bian, 2017; Editorial Committee of The Mirror Elucidating the way of Medicine, 2022).<br>The development of TCM in Hong Kong in the early 20th century (1900-1930): The British government suppressed TCM development and aimed to abolish TCM gradually (Bian, 2017; Editorial Committee of The Mirror Elucidating the way of Medicine, 2022).<br>The TCM in Hong Kong before the second World War and during the period occupation by Japan (1930 - 1945): Since the British took over Hong Kong, the government initially had no intention of dealing with Chinese affairs, so it allowed CM to be practiced in Hong Kong (Editorial Committee of The Mirror Elucidating the way of Medicine, 2022). Practicing CM only requires obtaining a commercial license to open a clinic. Citizens can only seek help from private CMPs with lower consultation fees, which allowed CM to develop under official pressure (Editorial Committee of The Mirror Elucidating the way of Medicine, 2022).<br>During 25 December 1940 to 15 August 1945, Japan occupied Hong Kong, and implemented the "Nationalization Movement" in Hong Kong, eliminating the colonial colour of British rule and suppressing the development of TCM" (Editorial Committee of The Mirror Elucidating the way of Medicine, 2022).<br>The TCM in Hong Kong after the Second World War until the 50 years of the 20th century (1945-1960): After Japan's unconditional surrender in August 1945, Hong Kong was taken over by the British on September 1 (Editorial Committee of The Mirror Elucidating the way of Medicine, 2022). The British government made no significant changes to CM policies which instead provided a favourable environment for the free development of private CM.<br>The TCM in Hong Kong during the 50 years to 70 years of the 20th century (1950-1980): After 1949, many mainlanders fled to Hong Kong, and doctors from all over the mainland followed suit and moved south to develop in Hong Kong (Editorial Committee of The Mirror Elucidating the way of Medicine, 2022). The local CM force rose rapidly and was roughly divided into the Guangdong school and the Northern school. In the era of "a hundred schools of thought contend", doctors not only treated patients but also diversified their development, including establishing guilds, running schools, publishing books, providing free	al context

		clinics, donating medicine, and engaging in international exchanges (Editorial Committee of The Mirror Elucidating the way of Medicine, 2022).<br>The TCM in Hong Kong since the 80 years of the 20th century (1980-1998): During the 1980s and 1990s, the Hong Kong industry actively participated in academic conferences held worldwide. It also had multiple opportunities to host international academic exchanges and organize various types of conferences (Editorial Committee of The Mirror Elucidating the way of Medicine, 2022). These activities demonstrate that the CM industry in Hong Kong has gained a certain level of international recognition and is acknowledged by industry professionals across Asia" (Editorial Committee of The Mirror Elucidating the way of Medicine, 2022).<br>After the reversion of sovereignty, CM was recognized by Hong Kong Special Administration Region Government and started the procedure of rapid development (Bian, 2017; Editorial Committee of The Mirror Elucidating the way of Medicine, 2022). The government was determined to develop CM and established CM clinics and hospitals. Tertiary institutions also began offering degree programs in CM. All these measures have brought about tremendous changes in the development of local CM.	
**Epidemiological context**	This domain indicates the distribution of conditions or disease, the determinants of demands, the attributable disease burden in human population (Rychetnik et al., 2002), and demographics (Castro et al., 2004; Hage et al.,	**Meso context**<br>• **Population:** Hong Kong SAR is a city occupying a 1,104-square-kilometer territory in China, where had a population of 7.4981 million residents from various nations (92% Chinese ethnicity) at mid-2023 (Census and Statistics Department, the Government of the Hong Kong Special Administrative Region, 2023a). Depression is a public health issue in Hong Kong. In 2019, the figure of prevalence of depression increased obviously to 11.2% (Ni et al., 2020) and jumped to 14.8%~19% in 2020 because of the effects of the coronavirus disease 2019 (Choi et al., 2020; Zhao et al., 2020).<br>• **Lifestyle:** lifestyle factors have been acknowledged to be modifiable targets that can be applied to counter the rising prevalence of depression (George et al., 2022; Zhao et al., 2023). Underlying neurobiological mechanisms involving brain structure, lifestyle, genetics, immunometabolic function and depression have been revealed (Zhao et al., 2023). Adhering to a healthy lifestyle could help in preventing depression (Zhao et al., 2023).	Socio-cultural context

	2013).		
**Geographical context**	The geographical characteristics mean the landscapes, wider physical surroundings, and resources, both natural and transformed by human beings, available at a given location.	**Macro (national level)**<br>• **A country with widely varying landscapes:** China, country of East Asia, stretches 5,000 kilometres from east to west and 5,500 kilometres from north to south. This large country with widely varying landscapes and its territory incorporates high plateaus, mountains, dense forests, and sandy deserts. China's diverse habitats are home to hundreds of species of plants and animals. For example, over 3,800 species of fish and hundreds of retile species and amphibians reside in the lakes, rivers, and coastal waters (National Geographic kids, 2024).<br>• **Meso (City level)**<br>**Location and climate suitable for TCM development in Hong Kong:** Hong Kong is located in the southern coastal region of China, surrounded by the sea on three sides. It has a typical maritime subtropical monsoon climate and a highland temperate monsoon climate. The weather is relatively humid and hot, which makes it rich in produce such as vegetables, fruits, and seafood. This natural background has given rise to local ethnic groups and culinary culture. Meanwhile, due to humid climate, Hong Kong locals people often drink soups and herbal teas to which they add cooling medicinal herbs in moderate amounts. This practice helps to dispel the dampness and heat, and to cool the body and prevent the common cold (Editorial Committee of The Mirror Elucidating the way of Medicine, 2022; Intangible cultural heritage office, 2023).	Socio-economic context
**Setting**			
**Setting context**	The setting includes the immediate organisational and physical environment, where an intervention is undertaken (Pfadenhauer et al., 2015; Pfadenhauer et	**Micro (research setting):** The research setting for conducting the TCMLM program was in a TCM course in School of Nursing, The Hong Kong Polytechnic University.<br>• **The Hong Kong Polytechnic University:** the university is located at the heart of Hong Kong, centrally situated in Hung Hom, Kowloon. The university campus covers around 9.46 hectares, and several choices of transportation services are available (e.g., Mass Transit Railway, bus, and Taxi) which makes it convenient to get to the Campus. The university is one of the prestigious universities locally and internationally. The university has been recognized as one of eight government-funded degree-granting tertiary institutions in Hong Kong and consistently ranked among the top 100 universities worldwide (The Hong Kong Polytechnic University, 2024). The school of nursing of the university is also very famous, ranking first in Hong Kong SAR and 21 globally according to the Shanghai Ranking's Global Ranking of Academic Subjects 2022 (Shanghai Ranking, 2024). The university, including school of nursing of the university, has a good reputation among Hong Kong residents, which may enhance the trust of local people towards courses provided by the university.<br>• **The research setting:** the setting for undertaking TCMLM program (except one session of wave 4 delivered via zoom video	Socio-cultural context and ethical context

	al., 2017). It also includes the effect the location has on affected stakeholder, such as by playing on a particular role (Pfadenhauer et al., 2015; Pfadenhauer et al., 2017).	communications because of Super Typhoon Saola, September 2, 2023) was in the Yuen Yuen Institute Chinese Medical Nursing Laboratory (圓玄學院中醫護理實驗室) (FJ501), which was in F building (陳大河樓) of the university. The FJ501 is a specialized laboratory designed for TCM nursing and is equipped with a pulse collector, pulse model, and multi-media acupuncture model. The facilities in FJ501 offer a clear concept of preliminary diagnosis, pulse pattern, and basic knowledge of acupuncture points in CM. The design of the laboratory and all internal facilities and decoration displayed in FJ501 conveyed the strong atmosphere of TCM, which may help participants to engage in sessions of TCMLM for depression better. Similarly, the Herbal Medicine Learning Centre, located in A110 of A building (鐘士元樓), has strong ambiance of TCM. The A110 was used as a venue for providing some sessions of TCMLM for depression for those participants who have been allocated in the waitlist control as compensations. Both A110 and FJ501 have adequate seating.	

Note. CM = Chinese medicine; CMCTRs = Chinese medicine clinics cum training and research centres; CMPs = Chinese medicine practitioners; GDP = gross domestic product; Hong Kong SAR = Hong Kong Special Administrative Region; ICD-11 = international statistical classification of diseases, 11th revision; ISO/TC = international organization for standardization/technical committee; ICWM = integrated Chinese-western medicine; TCM = traditional Chinese medicine; TCMLM = traditional Chinese medicine health lifestyle medicine; WHO = world health organization.

Table S3. Demographic characteristics of CMPs delivering the multicomponent TCM lifestyle medicine program for depression.

CMPs	Age	Gender	Education level	Years of working as Hong Kong CMP	Working units
CMP1	43	Male	Master's degree	21	University affiliated clinics
CMP2	32	Female	Doctorate degree	6-10	Private clinics
CMP3	36	Female	Master's degree	6-10	Hospital authority tripartite Chinese Medicine clinics

Note. CMP = Chinese medicine practitioner; TCM = traditional Chinese medicine.

Table S4. Demographic characteristics of five people with depression contributing to the program refinement (n = 5).

Participant	Age	Gender	Education level	Profession	Marital status	Depression diagnosed	Antidepressants taken
Volunteer 1	21	Male	Tertiary or above	University student	Single	Yes	Fluoxetine 20 mg/day
Volunteer 2	37	Male	Tertiary or above	Skilled worker	Single	Yes	No
Volunteer 3	59	Female	Secondary education	Professionals or semi-professionals	Single	Yes	No
Volunteer 4	20	Female	Tertiary or above	University student	Single	Yes	Paroxetine 30mg/day
Volunteer 5	20	Female	Tertiary or above	University student	Single	Yes	Dysthymia, Zoloft 25 mg/day

Table S5. Logic model review checklist for the multicomponent traditional Chinese medicine lifestyle medicine for depression.

Posing question quality criteria	Yes
Item 1: Problem statement	
1.1 The problem statement is a short description of the issue or problem that your program or service is designed to address.	
1.1.1 The problem is not framed as the lack of the service or program.	✓
1.1.2 The problem statement addresses the causes, not symptoms, of problems.	✓
1.2 The problem statement includes the following:	
1.2.1 Why does the problem exist?	✓
1.2.2 Who is affected by the problem?	✓
1.2.3 Who has a stake in solving the problem?	✓
1.2.4 What can be changed?	✓
Item 2: Objective	
2.1 The objective is a short description of what the program or service aims to achieve.	
2.1.1 Does the objective reflect other parts of the logic model? (e.g., Can you see how the activities contribute to the objective?)	✓
Item 3: Inputs	
3.1 Inputs are the resources that are required to deliver your program or service.	
3.1.1 Do the resources seem comprehensive?	✓
3.1.2 Do the inputs match the activities and outcomes?	✓
3.2 Are all the major resources listed? For example:	
3.2.1 service providers (e.g. staff, volunteers)	✓
3.2.2 partner agencies or other groups that will provide support or other in-kind contributions	✓
3.2.3 funding sources including: government, philanthropic, fee-for-service, donations	✓
3.2.4 research or evidence base, including program manuals or packages	✓
Item 4: Outputs: Activities	
4.1 Activities are the things that you do in your program. For example: delivering group counselling sessions or mindfulness activities.	

4.1.1 Are all the major activities listed?	✓
4.1.2 Is there a sufficient level of detail to understand what is delivered as part of the program (and how and to who)?	✓
4.1.3 Do the activities seem sufficient to achieve the outcomes?	✓
4.1.4 Do the activities match good practice in addressing the problem (as identified in the problem statement)?	✓
4.2 The participation column describes the intended participants or clients for your program or service, and any inclusion or exclusion criteria.	
4.2.1 Are the intended participants clearly defined?	✓
4.4.2 Are all audiences for the program or service included?	✓
4.4.3 Are the mix and intensity of activities appropriate for the intended participants?	✓
Item 5: Outcomes	
5.1 The short, medium, and long-term outcomes describe the impact of your program or service on the participants, families and communities that you are working with.	
5.1.1 Are the outcomes truly outcomes (and not outputs)?	✓
5.1.2 Are the outcomes measurable? Will you be able to measure if the outcome has been achieved?	✓
5.1.3 Are the outcomes logically connected – meaning that one can be expected to lead to another – and supported by evidence?	✓
5.1.4 Are the outcomes realistic? Are they likely to occur given the inputs and activities, and the scale of the problem?	✓
5.1.5 Are the time frames for the outcomes achievable?	✓
5.1.6 Are the outcomes likely to have an effect on the problem in the problem statement?	✓
5.1.7Are the outcomes meaningful to the participants?	✓
5.1.8 Are the outcomes written as change statements? Will things increase, decrease, or stay the same?	✓
Item 6: Assumptions	
6.1 Assumptions are the conditions surrounding a program or service that need to be in place for the program or service to work as intended (e.g. the program will be accessible for participants, staff will implement the program material).	
6.1.1 Have you asked other people involved with the program or service to identify assumptions?	✓
6.2 Have you considered assumptions in relation to:	
6.2.1 the problem?	✓
6.2.2 the resources?	✓

6.2.3 staff (e.g. retention rates or having adequately trained staff)?	✓
6.2.4 the activities?	✓
6.2.5 participants?	✓
6.2.6 the way outcomes connect to each other?	✓
Item 7: External factors	
7.1 External factors are economic, political, cultural, historical, and social factors that affect the way a program is delivered and the outcomes that can be achieved.	
7.1.1 Have you asked other people involved with the program or service to identify external factors that might affect program outcomes?	✓
7.2 Have you considered external factors in the following areas?	
7.2.1 economic	✓
7.2.2 political	✓
7.2.3 cultural	✓
7.2.4 historical	✓
7.2.5 social	✓
7.2.6 organizational	✓
Item 8: Overall review questions	
8.1 These are questions to ask once you are happy with the individual elements of your program logic.	
8.1.1 Is the model truly logical? Do the inputs, outputs and outcomes lead on from one to another and make sense?	✓
8.1.2 What might be the unintended or negative outcomes of the program?	✓
8.1.3 Can the program logic be understood by someone unfamiliar with the program?	✓
8.1.4 Can the program, as described in the logic model, be implemented with available resources?	✓

Note. Adapted from *Developing a Logic Model: Teaching and Training Guide* (p. 49-50), by E. Taylor-Powell and E. Henert, 2008, University of Wisconsin-Extension. Copyright 2008 by the Board of Regents of the University of Wisconsin System (https://fyi.extension.wisc.edu/programdevelopment/files/2016/03/lmguidecomplete.pdf). Reprinted with permission.

Table S6. The median, IQR, and percentage agreement values in round 1 Delphi survey.

Items	Median	IQR	Percentage agreement (scoring 6/7 or 7/7)
Section 1: The curriculum setting of *nourishing the heart*			
1. Introduce the definition of TCM health preservation	6.5	1.00	100%
2. Introduce the theoretical foundation of TCM health preservation	7.0	1.25	80%
3. Introduce the nine dimensions of TCM health preservation: nourishing the heart, nourishing the *Qi*, food tonics for health preservation, conducting exercises, nourishing according to time, nourishing during old age, nourishing during the pregnancy, herbs for health preservation, and sexual hygiene	6.5	2.00	70%
4. Introduce the etiology and pathogenesis of TCM health preservation and emotion (depression)	7.0	1.00	90%
5. Introduce the syndrome differentiation and treatment of TCM health preservation and emotion	6.5	1.00	90%
6. Introduce the therapeutic strategies of TCM health preservation and emotion, including nourishing the heart, nourishing the *Qi*, and nourishing according to time	7.0	1.00	90%
7. Define mental health care in nourishing the heart	6.0	2.00	70%
8. Describe the specific method of mental health care in nourishing the heart: Distraction	6.0	1.50	80%
9. Describe the specific method of mental health care in nourishing the heart: Implicit method	6.0	2.25	60%
10. Describe the specific method of mental health care in nourishing the heart: Emotional modulation based on the five elements generated and counterbalance with each other laws	6.5	2.00	70%
11. Describe the specific method of mental health care in nourishing the heart: Enlightened method	6.0	1.00	90%
12. Describe the specific method of mental health care in nourishing the heart: Temperance method	6.0	1.00	90%
13. Describe the specific method of mental health care in nourishing the heart: Catharsis method	6.0	1.00	90%
14. Describe the specific method of mental health care in nourishing the heart: Spiritual cultivation in the four seasons	7.0	1.25	80%
15. Describe the specific method of mental health care in nourishing the heart: Abstinence and essence conservation, clear heart, and calm mind	6.5	1.25	80%
16. Describe the specific method of mental health care in nourishing the heart: *Qi* regulation	6.5	1.00	90%
17. Practice *Baduanjin*, a method of Qi regulation, in class with the instructor	6.5	1.00	100%
18. Describe the specific method of mental health care in nourishing the heart: Meditation	7.0	1.25	80%

19. Practice meditation, a method of mental health care in class	7.0	1.25	80%
20. Provide simple, syndrome differentiation-based diet suggestions for depression in class	6.5	1.00	90%
21. Define refined hobbies in nourishing the heart	6.5	1.00	90%
22. Describe a refined hobby for nourishing the heart: Music therapy	7.0	1.00	90%
23. Try a refined hobby by listening to a Chinese song that could nourish the heart	6.0	2.25	60%
24. Try a refined hobby by listening to a foreign song that could nourish the heart	6.0	2.25	60%
25. Describe a refined hobby in nourishing the heart: Reading beautiful articles	6.0	1.50	80%
26. Try a refined hobby by reading a beautiful article that could nourish the heart	6.0	1.50	60%
27. Describe a refined hobby for nourishing the heart: Painting and calligraphy	6.0	2.00	70%
28. Try a refined hobby by appreciating a beautiful painting and/or calligraphy that could nourish the heart	6.0	2.00	70%
29. Describe a refined hobby for nourishing the heart: Playing chess	6.0	1.50	80%
30. Describe a refined hobby for nourishing the heart: Angling	6.0	2.00	60%
31. Describe a refined hobby for nourishing the heart: Appreciation of flowers and birds	6.0	1.00	90%
32. Describe a refined hobby for nourishing the heart: Travel	6.5	1.00	90%
33. Describe a refined hobby for nourishing the heart: Tasting tea	7.0	1.00	90%
34. Describe a refined hobby for nourishing the heart: Collecting	6.0	3.00	60%
35. Describe a refined hobby for nourishing the heart: Aromatherapy	6.5	1.25	80%
36. Describe a refined hobby for nourishing the heart: Watching Chinese opera and singing songs	6.0	1.50	60%
37. Describe a refined hobby for nourishing the heart: Swimming and bathing	5.5	1.50	50%
38. Describe a refined hobby for nourishing the heart: Species reintroduction	4.0	5.00	30%
39. Describe the definition and function of social interaction in nourishing the heart	6.0	1.00	100%
40. Describe the principles and measures of establishing a healthy communicative environment in social interaction for nourishing the heart	6.0	1.25	80%
Section 2: The curriculum setting of *nourishing according to time*			
1. Describe the concepts and principles of health-preserving daily schedule, which includes conforming to the laws of nature, complying with the patterns of the human body. and maintaining the moderation of works and rests	7.0	1.00	100%
2. Describe the TCM theories and knowledge in sleep, including sleep stages, the role of sleep, and the judgment of sleep quality, etc.	6.5	1.00	100%
3. Describe the "*Ziwuliuzhu*" (midnight-noon ebb-flow) method in sleep hygiene, a sleep regulation method	5.5	3.25	50%
4. Describe self-regulating sleep aids (meditation, self-hypnosis to fall asleep, tranquility)	7.0	1.25	80%

5. Describe 3–4 acupoints that can improve sleep quality, relieve depression, and be easily manipulated [Baihui (DU20), Shenmen (HT7), Neiguan (PC6), Yintang (EX—HN3)]	7.0	1.00	90%
6. Learn and practice acupoints that can improve sleep quality and relieve depression in class, including [Baihui (DU20), Shenmen (HT7), Neiguan (PC6) and Yintang (EX—HN3)]	7.0	1.00	90%
7. Describe sleep regulation methods, including "regulation before bedtime," "regulation at bedtime," and "ten contraindications regarding sleep"	6.0	1.25	80%
8. Describe a sleep aid, diet for better sleep; for example, taking a small amount of food for better sleep, such as walnut, honey, lily, longan, milk, and coix seed	6.0	2.00	70%
9. Describe a sleep aid, shen-calming music	6.5	1.00	90%
10. Close the eyes and listen to calming music to experience nourishing according to time in class (e.g., five tone music)	6.5	1.25	80%
11. Describe a sleep aid, Traditional Chinese Medicine aromatherapy (e.g., lavender, orange peel, and rose)	5.0	3.00	40%
12. Practice nourishing according to time through tasting shen-calming foods and drinks, such as herbal tea, in class	7.0	2.00	70%
Section 3: The curriculum setting of nourish*ing the Qi*			
1. Define and describe the function of *nourishing the Qi*	7.0	1.00	100%
2. Describe the relationship between "nourishment Qi" and "depression" in TCM ("adjustment of body," "regulation of breathing," "regulation of mental activities")	7.0	1.00	100%
3. Describe the philosophical foundation of Qigong in nourishing the *Qi*	6.0	1.00	90%
4. Describe the relationship between Qigong and depression and the related function of Qigong on depression	6.0	1.00	100%
5. Describe diet suggestions related to TCM emotion and depression and nourishing the *Qi*	6.0	2.00	70%
6. Describe *Baduanjin*, a method for *nourishing the Qi*, including overview, function, and practical method	7.0	1.00	100%
7. Experience nourishing the *Qi* by learning and practicing *Baduanjin* in class (twice)	6.0	1.00	100%
8. Describe six-character formula, a method for nourishing the *Qi*, including overview, function, and practical method	7.0	1.00	90%
9. Experience nourishing the *Qi* by learning and practicing six-character formula in class (twice)	6.5	1.25	80%

Note. IQR = interquartile range; TCM = traditional Chinese medicine.

Table S7. Fourteen items removed in Round 2 and related reasons.

Sections	Items	Reasons of choosing each question on "strongly disagree," "disagree," or "disagree somewhat"
Section 1: The curriculum setting of *nourishing the heart*		
	1. Introduce the nine dimensions of TCM health preservation: nourishing the heart, nourishing the *Qi*, food tonics for health preservation, conducting exercises, nourishing according to time, nourishing during old age, nourishing during the pregnancy, herbs for health preservation, and sexual hygiene	Some content irrelevant to depression; Not fit in modern society; Lacking reliable references
	3. Describe the specific method of mental health care in nourishing the heart: Implicit method	Difficulty in practice or show in class; Doubt potential efficacy
	4. Describe the specific method of mental health care in nourishing the heart: Emotional modulation based on the five elements generated and counterbalance with each other laws (examples)	Not fit in modern society; Doubt potential efficacy
	5. Try a refined hobby by listening to a Chinese song that could nourish the heart	Songs should be based on five tone music but may not fit in modern society
	6. Try a refined hobby by listening to a foreign song that could nourish the heart	Songs should be related to five music and five elements: Unfamiliar with music
	7. Describe a refined hobby in nourishing the heart: Reading beautiful articles	Not suitable to practice in class; Not fit in the fast-pace modern life
	8. Describe a refined hobby for nourishing the heart: Painting and calligraphy	Influence nourishing the heart negatively due to inappropriate painting theme
	10. Describe a refined hobby for nourishing the heart: Angling	Difficulty in practice or show in class; Not fit in fast-pace modern life; be harmful to fish.
	11. Describe a refined hobby for nourishing the heart: Collecting	Difficulty in practice or show in class; An expensive activity
	12. Describe a refined hobby for nourishing the heart: Watching Chinese opera and singing songs	May negatively be influenced by Chinese opera, leading to an emotional state.

13. Describe a refined hobby for nourishing the heart: Swimming and bathing	Difficulty in practice or show in class.
14. Describe a refined hobby for nourishing the heart: Species reintroduction	Difficulty in practice or show in class; Damage the ecosystem.
Section 2: The curriculum setting of nourishing according to time	
1. Describe“*Ziwuliuzhu*” (midnight-noon ebb-flow) method in sleep hygiene, sleep regulation method	Still disputed content in academic
4. Describe a sleep aid, TCM aromatherapy (e.g., chamomile, lemon, or its mixture)	Not related to TCM (e.g., chamomile, lemon, or its mixture); Aromatherapy not belonging to TCM health preservation method

Note. TCM = traditional Chinese medicine.

Table S8. The median, IQR, and percentage agreement values in round 2 Delphi survey.

Sections	Items	Median	IQR	Percentage agreement (scoring 6/7 or 7/7)
Section 1: The curriculum setting of *nourishing the heart*				
	1. Introduce the nine dimensions of TCM health preservation: nourishing the heart, nourishing the Qi, food tonics for health preservation, conducting exercises, nourishing according to time, nourishing during old age, nourishing during the pregnancy, herbs for health preservation, and sexual hygiene	6.0	1.50	70%
	2. Define mental health care in nourishing the heart	7.0	1.0	90%
	3. Describe the specific method of mental health care in nourishing the heart: Implicit method	5.5	3.25	50%
	4. Describe the specific method of mental health care in nourishing the heart: Emotional modulation based on the five elements generated and counterbalance with each other laws (examples)	6.0	3.00	60%
	5. Try a refined hobby by listening to a Chinese song that could nourish the heart	6.0	2.25	60%
	6. Try a refined hobby by listening to a foreign song that could nourish the heart	6.0	2.00	70%
	7. Describe a refined hobby in nourishing the heart: Reading beautiful articles	6.0	1.50	60%
	8. Describe a refined hobby for nourishing the heart: Painting and calligraphy	6.0	1.00	60%
	9. Try a refined hobby by appreciating a beautiful painting and/or calligraphy that could nourish the heart	6.0	1.25	80%
	10. Describe a refined hobby for nourishing the heart: Angling	6.0	3.00	60%
	11. Describe a refined hobby for nourishing the heart: Collecting	6.0	3.25	60%
	12. Describe a refined hobby for nourishing the heart: Watching Chinese opera and singing songs	5.5	1.50	50%
	13. Describe a refined hobby for nourishing the heart: Swimming and bathing	5.0	2.25	40%
	14. Describe a refined hobby for nourishing the heart: Species reintroduction	3.5	4.25	30%
Section 2: The curriculum setting of *nourishing according to time*				
	1. Describe the "*Ziwuliuzhu*" (midnight-noon ebb-flow) method in sleep hygiene, a sleep regulation method	6.0	3.25	60%
	2. Describe a sleep aid, diet for better sleep; for example, taking a small amount of food for better sleep, such as lily, longan, and jujube	6.0	1.00	90%
	3. Describe the principle of eating according to time and season, and introduce seasonal food	6.0	1.25	90%
	4. Describe a sleep aid, TCM aromatherapy (e.g., lemon, or its mixture)	4.0	3.50	30%
	5. Practice nourishing according to time through tasting shen-calming foods and drinks, such as dried longan and sour jujube in class	6.0	0.75	80%

Section 3: The curriculum setting of *nourishing the Qi*			
1. Describe diet suggestions related to TCM emotion and depression and nourishing the *Qi*	6.5	1.00	100%
2. Describe basic techniques of simple qigong, a method for nourishing the *Qi*, including overview, function, and practical method	7.0	1.00	100%
3. Experience nourishing the *Qi* by learning and practicing simple basic techniques of Qigong in class (twice)	6.0	1.00	90%

Note. IQR = interquartile range; TCM = traditional Chinese medicine.

Table S9. Rank of dimensions for development of multiconponet traditional Chinese medicine lifestyle medicine program for depression.

Rank	Items	Scores
1	Theoretical knowledge of Traditional Chinese Medicine health preservation	37
2	Theoretical knowledge of mental health preservation	28
3	Theoretical knowledge of hobby for nourishing the heart	21
4	Theoretical knowledge of social interaction	14

Table S10. Examples of content revised or added to fit the Hong Kong context.

Context revised or added during the Delphi survey			Context revised or added based on evidence reviewing	
Content	Strategies	Reasons	Content and strategeis	Reasons
"Describe a refined hobby for nourishing the heart: Angling" under component 1	Removed in Round 2 Delphi suvery	Difficulty in practice or showing in class, not fitting in fast-pace modern life in Hong Kong and bringing harm to fish.	The "date, bamboo, and heart of the lamp porridge" (Jujube seeds [20 g], polygonatum rhizome [20 g], heart of the lamp grass [6 g], and glutinous rice [200 g]) and the "donkey-hide gelatin and Buddha's hand citron soup (donkey-hide gelatin [5 g], dried Buddha's hand citron peel [10 g], arborvitae seed [15 g], chicken liver [1 piece], and rock sugar [20 g]), were replaced by "fresh mushroom fish slices" (fresh shiitake mushrooms [200 g] and grass carp meat [100 g])	The Cantonese cuisine in Hong Kong puts emphasis on applying seasonal and fresh ingredients, having a diverse range of seafood, being known for its more delicate and lighter flavors, being famous for a wide variety of dim sum options (often served in small parts), and using steaming and stir-frying skills (roasting, slow braising, deep-frying methods in Guangdong cuisine). Therefore, some cuisine recipes with benefits of shen-calming and improving sleep quality were replaced by localized cuisine recipes with similar functions.<br>In this alternative recipe, grass carp, is adopted. Grass carp, well known for its flavorful and tender meat, is a popular fish enjoyed by many people in Hong Kong. In this region, grass carp can be prepared in different ways, incorporating steaming, pan frying, or simmering in soups.
"Describe basic techniques of simple qigong, a method for nourishing the Qi, including overview, function, and practical method" under component 3	Incorporated in Round 2 Delphi suvery	The high possibility of the majory of Hong Kong adults lacking basic knowledge regarding Qigong.		

Table S11. Examples of comments during the iterations of refinement and relevant modifications for multicomponent TCM lifestyle medicine program for depression.

Number	From research team members and those who would deliver the program		From five participants with depression	
	Comments	Relevant modifications	Comments	Relevant modifications
Comment 1	Not suitable expression as "mania" for *excessive yang* and "listlessness" for *excessive yin*	Modifying to present as "excessive yang leading to being hyperactive and restless" and "excessive ying leading to feeling down"	Not understanding some terminologies and not sure about the relationship between *nourishing the heart* and TCM health preservation	(1) Providing the definitions of TCM health preservation, *nourishing the heart*, *nourishing according to time*, and *nourishing the Qi* in the manual of TCM health preservation program for depression; and (2) Training CMPs to introduce the definitions and relationships in sessions related
Comment 2	Suggesting adding two TCM terminologies under the etiology and pathogenesis of TCM health preservation, namely, the six external factors of disease (Six Yin) and the seven emptions (Qi Qing)	The two TCM terminologies have been added into the teaching materials of the session 1 of *nourishing the heart*	Not quite understanding some TCM terminologies like "ascending ad descending" (*升降沉浮* in Chinese), "returning to the meridian" (*歸經* in Chinese)	(1) Adding more information regarding these terminologies in the teaching materials, such as definitions and examples of ingredients related to certain meridian like "black sesame tonifying kidney"; and (2) Training CMPs to explain in detailed regarding these terminologies while providing relevant sessions
Comment 3	Providing the rough time regarding how long before the bedtime not drinking and consuming food	Adding information showing as "not drinking and consuming food one hour before bedtime"	Suggesting providing more Chinese dietary therapy recipes, especially those for different seasons	Adding more examples of seasonal food based on different seasons in the teaching materials of *nourishing according to time*

Comment 4	The two Chinese dietary therapy, the "Date, bamboo, and heart of the lamp porridge" and the "Donkey-hide gelation and buddha's hand citron soup", not well consistent with Hong Kongers' eating habits and not healthy and delicious	Replying the two not localized Chinese dietary therapies by the "Fresh mushroom fish slices"	Being better to introduce the negative effects of bad sleeping positions	Providing the CMPs regarding the negative effects of bad sleep positions and training them to provide related information in the relevant session
Comment 5	Suggesting providing the information of the nature, taste, returning to meridians, and functions of tangerine peel as well as the method for making tangerine peel drink	Adding information of nature, taste, returning to meridians, and functions of tangerine peel as well as the method for making tangerine peel drink	No explanations regarding the Five Elements complementing each other	Providing detailed information regarding the Five Elements complementing each other to CMPs and training them to provide the information while delivering the relevant session

Note. CMP = Chinese medicine practitioner; TCM = traditional Chinese medicin

Appendix G

Program Manual: Multicomponent Traditional Chinese Medicine Lifestyle Medicine Program for Depression Manual

Institution Affiliation:

School of Nursing,

the Hong Kong Polytechnic University

Date of Publication: May 2023

List of Contents

Section 1 Introduction

1.1 Background and rationale

1.2 Program objectives and anticipated outcomes

1.3 Target population and participants

1.4 Scope and structure of the manual

Section 2 Program overview

2.1 Program description

2.2 Key components and sessions

2.3 Mode, format, frequency, duration, and timeline

2.4 Roles and responsibilities of stakeholders

Section 3 Program theory and logic model for the program

3.1 Program theory for the program

3.2 Logic model for the program

Section 4 Program delivery and implementation

4.1 Program providers and venues

4.2 Program component and sessions

4.3 Program materials

Section 1 Introduction

1.1 Background and rationale

Depression refers to a debilitating mental disorder with unknown causes (APA, 2013). During a depressive period, an individual suffering from depression may experience a series of symptoms, such as depressed mood, a loss of pleasure or interest in activities, feeling worthless or guilty, resulting in significant impacts on mental well-being, physical health, social functioning, and economic burden. Depression can be treated by two mainstream methods, cognitive behavioral therapy, and antidepressants, however, the health-seeking behavior for treating depression is low (Wong et al., 2023) and the depression treatment status is dissatisfactory (Patel et al., 2018), which might be related to the side effects of using medications and depression-related stigma. Integrative lifestyle medicine, one type of complementary and alternative medicine, has been shown its effectiveness for treating depression (Wong et al., 2021).

Multicomponent TCM lifestyle medicine, also known as TCM health preservation (*Yang-sheng, 中醫養生 in* Traditional Chinese), which shares similar principle like lifestyle medicine, might be a potential novel less-stigmatized treatment for depression, especially for those individuals with depression living in countries or regions in Eastern Asia where the TCM originated in (China) or spread to (e.g., Japan, Korea, Taiwan). Hong Kong, a Special Administrative Region of the People's Republic of China, is just as such kind of region. As the number of people living with depression continues to rise in Hong Kong (Choi et al., 2020; Ni et al., 2020; Wong et al., 2022), a novel treatment for the mental illness is warranted. Therefore, it is necessary to develop TCM health preservation program for depression, and implement the program, therefore research-based evidence might generate to support the feasibility, acceptance, and preliminary effects of TCM health preservation program for depression. A program manual is the key part regarding program implement, therefore, we design and report the multicomponent TCM lifestyle medicine program manual herein.

1.2 Program objectives and anticipated outcomes

The objectives and anticipated outcomes of the multicomponent TCM lifestyle medicine program for depression are described as follows.

1.2.1 Key objectives

Objective 1: To educate participants about the impact of multicomponent TCM lifestyle medicine

consisting of *nourishing the heart*, *nourishing according to time*, and *nourishing the Qi* on depression and other health-related outcomes (anxiety, stress, fatigue, sleep quality, quality of life, health-promoting lifestyle behavior) through context-based, theory-based, evidence-based, and less-stigmatized program.

Objective 2: To increase motivation of practicing multicomponent TCM lifestyle medicine and to facilitate TCM lifestyle habit formation among participants by distributing printed teaching materials, allocating logbooks, tasting herbal tea drink at sessions, and providing herbal tea packet as souvenir.

Objective 3: To improve participants' adherence to learn and practice the multicomponent TCM lifestyle medicine by applying interactive techniques, motivational interviewing strategies, teach-back method, and showing empathy and encouragement.

Objective 4: To promote collaboration and partnerships with key stakeholders, research team members, and program providers who are local Chinese Medicine practitioners (CMPs) to enhance program reach, sustainability, and effectiveness.

Objective 5: To evaluate the feasibility, acceptance, and preliminary efficacy of multicomponent TCM lifestyle medicine program for depression and other health-related outcomes through feasibility evaluation, acceptance evaluation, outcome evaluation and process evaluation.

1.2.2 Anticipated outcomes

Outcome 1: Significant reduction in depressive symptoms and improvement in other health-related outcomes as all measured by standardized assessment instruments (Patient Health Questionnaire-9 for depression, Generalized Anxiety Disorder-7 for anxiety, Perceived Stress Scale for stress, Fatigue Assessment Scale for fatigue, Insomnia Severity Index for sleep quality, Short Form 6-Dimension for quality of life, and Health-Promoting Lifestyle Profile II for health-promoting behaviors).

Outcome 2: Enhanced awareness, knowledge, skills, and confidence among participants in adopting and maintaining multicomponent TCM lifestyle medicine, leading to reduced depression and improved health-related outcomes.

Outcome 3: An interactive, dynamic, empathic, supportive, and encouraging program, fostering a sense of belonging, and understanding, which both promote TCM lifestyle practice.

Outcome 4: A collaborative network of partnerships with key stakeholders, research team members, and CMPs, which will foster shared responsibility.

Outcome 5: Comprehensive program evaluation and feedback insights, guiding continuous

improvement, and alignment with evidence-based practices, participant demands, and emerging research.

1.3 Target population and participants

The target population refers to Chinese adults aged 18 to 65 years suffering from moderate depression measured by the Patient Health Questionnaire-9 (PHQ-9) in Hong Kong (Kroenke et al., 2001). The detailed inclusion and exclusion criteria of the target population are described as follows.

The inclusion criteria: (1) Hong Kong Chinese adults aged 18-years-old to 65-years-old; (2) diagnosed with moderate level of depression (a score of 10~20), evaluated by the PHQ-9 (Kroenke et al., 2001); (3) can communicate in spoken Cantonese and written Chinese; and (4) willing to provide written informed consent and be able to adhere to the study protocol.

The exclusion criteria: (1) new onset or change of antidepressant medication or dosage in the last 3 months (i.e., the antidepressant therapy has to be at the fixed dose for at least 3 months, indicating a stable depressive state); (2) PHQ score≥20, meaning a severe level of depression (Kroenke et al., 2001); (3) with a previous or present diagnosis of psychotic diagnosis or serious psychiatric illness (e.g., schizophrenia, substance dependence or abuse, or bipolar disorder) as screened by the Chinese version of the Structured Clinical Interview for DSM-IV; (4) the score of Hong Kong Montreal Cognitive Assessment less than 22, showing that cognitive impairment that may prevent understanding of training instructions (Yeung et al., 2014); (5) being pregnant or during the lactation, or plan to get pregnant but not using adequate contraception; (6) significant suicidal risk as rated by the Hamilton Depression Rating Scale item on suicide (score ≥3) (Zheng et al., 1988); (7) with diagnosis of illness which can interfere the individuals to participate or adhere to the modified lifestyle, such as major neurocognitive disorders, medical disorders or psychiatric illness; (8) with diagnosis of medical conditions, which are not suitable to modify diet or do physical activities recommended by physicians; (9) joining in another clinical trial during the study period; and (10) with any major medical condition that causes depression based on the judgement of the investigator.

By contrast, the participants mean those individuals within the target population who will actively engage, enroll, or participate in the activities or services of the program. They are the direct beneficiaries or recipients of the multicomponent TCM lifestyle medicine program's initiatives.

1.4 Scope and structure of the manual

The program manual targets at equipping Chinese adults aged 18-65 years suffering from moderate depression measured by the PHQ-9 in Hong Kong with multicomponent TCM lifestyle medicine knowledge and skills to increase TCM lifestyle medicine awareness, improve their confidence of learning and practicing of multicomponent TCM lifestyle medicine, as well as enhance the practice and adherence of TCM lifestyle.

The structure of the program manual consists of nine sections. Section 1 introduction refers to four parts, background and rationale, program objectives and anticipated outcomes, target population and participants, as well as scope and structure of the manual. Section 2 program overview is composed of four parts, including program description; key components and sessions; mode, format, frequency, duration, and timeline; as well as roles and responsibilities of stakeholders. Section 3 program theory and logic model for the program just involves the two parts and is introduced separately in the manual. Section 4 program delivery and implementation incorporates four parts, namely, program providers and venues, program component and sessions, and program materials.

Section 2 Program overview

2.1 Program description

The multicomponent TCM lifestyle medicine program for depression is a comprehensive initiative designed to empower Chinese adults aged 18 to 65 years suffering from moderate depression in Hong Kong with the knowledge, skills, and resources necessary of multicomponent TCM lifestyle medicine to reduce their depressive symptoms and improved other health-related outcomes. In consideration of the common issue of low motivation and poor adherence to many depression programs, this program adopts a development process which is context-based, theory-based, evidence-based, and less stigmatized; and meanwhile, this program applies interactive techniques, motivational interviewing strategies and teach-back method, as well as showing empathy and encouragement.

2.2 Key components and sessions

There are three key components involving six sessions of the multicomponent TCM lifestyle medicine program for depression. Component 1: *nourishing the heart.* Component 2: *nourishing according to time*. Component 3: *nourishing the Qi.* Each component is delivered through two sessions, such as "*nourishing the heart* 1" and "nourishing the heart 2".

2.3 Mode, format, frequency, duration, and timeline

The multicomponent TCM lifestyle medicine program for depression program adopts face-to-face group sessions (mode and format) and will be held on a weekly basis (frequency). The overall program will span a duration of 6 weeks (overall program duration) and each session of the program will last for 2 hours (session duration).

When the 6-week multicomponent TCM lifestyle medicine program for depression sessions has been delivered, the participants will receive a 6-week follow-up period. If some participants could not attend the session in-person, audio recording of the session will be shared to those participants (virtual components; mode and format). If in-person sessions could not be held due to an unexpected situation (e.g., signal No.8 or above of tropical cyclone, black rainstorm, or other extreme conditions), the session will be held via zoom as virtual sessions.

Timeline for the 6-week multicomponent TCM lifestyle medicine program for depression incorporate:

Week 1: *Nourishing the Heart* 1

Week 2: *Nourishing the Heart* 2

Week 3: *Nourishing According to Time* 1

Week 4: *Nourishing According to Time* 2

Week 5: *Nourishing the Qi* 1

Week 6: *Nourishing the Qi* 2

2.4 Roles and responsibilities of stakeholders

2.4.1 Funders

Fund information: This research project is funded by one provincial level grant, the Chinese Medicine Development Fund of the Hong Kong Special Administrative Region Government (grant number: 21B2/004A_R1); and one university-level grant, the Department General Research Fund of the Hong Kong Polytechnic University (grant number: P0036822).

Role: provide grants to support the program's activities, initiatives, projects, and operations.

Responsibility: (1) allocate, disburse, and manage funds according to agreed-upon budgets, timelines, objectives, and reporting requirements; (2) collaborate with program stakeholders to ensure coherence, relevance, and synergy between funder priorities and program initiatives; (3) participate in meetings and discussions to exchange insights, updates, and recommendations related to program

implementation, challenges, opportunities, and innovations; (4) review program documentation, reports, data, insights, and feedback to inform funding decisions, adjustments, and strategic priorities; and (5) collaborate with program teams to adapt, refine, innovate, or scale initiatives, strategies, programs, and approaches in response to emerging challenges, needs, opportunities, and lessons learned.

2.4.2 Principal investigator (also as program coordinator)

Role: oversee the comprehensive planning, implementation, coordination, and evaluation of the multicomponent TCM lifestyle medicine program for depression

Responsibilities: (1) coordinate stakeholder meetings, communication, and collaboration; (2) develop program objectives, goals, and strategies with stakeholders; (3) monitor program progress, outcomes, and adherence to budgets and timelines; and (4) evaluate the feasibility, acceptance, and preliminary efficacy of the program, participants feedback, and stakeholders' contributions.

2.4.3 Mental health professionals (a clinical psychologist, a psychiatrist, and two mental health nurses)

Role: provide professional suggestions, guidance, and support during the design, development, and implementation of the program

Responsibilities: (1) collaborate with other stakeholders to develop a multicomponent TCM lifestyle medicine program effectively; (2) provide feedback and identify opportunities for continuous improvement.

2.4.4 Chinese medicine practitioners (program instructors)

Role: deliver a multicomponent TCM lifestyle medicine program for depression sessions, guidance, and support to empower participants in making lifestyle-related changes.

Responsibility: (1) facilitate sessions and activities focused on TCM lifestyle medicine; (2) guide participants set TCM lifestyle practice goals; (3) increase the participants' motivation for learn and practice TCM lifestyle, as well as adherence to practice TCM lifestyle medicine through applying interactive techniques, motivational interviewing strategies, teach-back method, and show empathy and encouragement; (4) monitor participants' engagement, progress, barriers, and facilitators while learning and practicing TCM lifestyle; (5) collaborate with other team members to ensure integrated multicomponent TCM lifestyle medicine program for depression delivered; and (6) reflect on delivering experiences, challenges, facilitators, and provide continuous improvement suggestions,

2.4.5 Participants

Role: Actively engage, participate, and collaborate in the multicomponent TCM lifestyle medicine program for depression to achieve TCM lifestyle goals as well as reduce depression and improve other health-related outcomes.

Responsibility: (1) attend the program sessions and actively participate in program activities; (2) implement strategies, skills, and recommendations provided by the program to promote TCM-based lifestyle practice; (3) set weekly TCM lifestyle goals, practice goals as far as possible and write down timely in the logbooks; (4) communicate needs, concerns, feedback and progress with stakeholders to receive support, guidance, and resources; and (5) complete assessment within the required time, report side effects timely.

Section 3 Program theory and logic model for the program

3.1 Program theory for the program

The program theory for the multicomponent TCM lifestyle medicine program for depression is developed based on theoretical source and empirical source. Theoretical source refers to some theoretical constructs from the Transtheoretical model, including self-revaluation, self-efficacy, helping relationships, reinforcement management, social liberation, consciousness raising, counter conditioning, and stimulus control (Prochaska & Prochaska, 2019); and is influenced by the stages of change of the Transtheoretical model (precontemplation, contemplation, preparation, action, and maintenance) (Prochaska & Prochaska, 2019). Empirical source is composed of reviewing of TCM health preservation literature and books (Ruan et al., 2023; Sun, 2017; Tan, 2009; Wang, 1992; Xu & Hu, 2016; Zhang, 2011), lifestyle medicine literature and books related to mental health (particularly in depression) (Chu et al., 2022; Edshteyn, 2019; Frates, & Eubanks, 2019; Gholami et al., 2019; Glanz et al., 2015; Ip et al., 2021; Lenz, 2014; Wong et al., 2021) as well as TCM-related clinical practice guidelines for depression (China Association of Chinese Medicine, 2015; Guo et al., 2008; Ma & Zhang, 2021; Societies, 2020; Zhao et al., 2010; Zhao et al., 2015).

3.2 Logic model for the program

Guided by the program theory, a logic model for the multicomponent TCM lifestyle medicine program for depression has been developed and has been shown in Fig. 2 in the manuscript. The logic model is on the basis of the premise that how individuals suffering from depression live the life can

impact their emotion and other health-related outcomes. Modifying unhealthy lifestyle in line with the TCM lifestyle medicine theory will lead to depression, anxiety, stress and fatigue reduction, as well as improvement of sleep quality, quality of life, and health-promoting lifestyle behavior.

Section 4 Program delivery and implementation

4.1 Program providers and venues

Program providers are Hong Kong Chinese Medicine practitioners. The venues for delivering the multicomponent TCM lifestyle medicine program for depression include FJ501 or A110, both classrooms belong to the School of Nursing, the Hong Kong Polytechnic University.

4.2 Program component and sessions

There are three key components involving six sessions of the multicomponent TCM lifestyle medicine program for depression. Component 1: *nourishing the heart.* Component 2: *nourishing according to time*. Component 3: *nourishing the Qi.* Each component will be delivered through two modules, such as "*nourishing the heart* 1" and "nourishing the heart 2". In each module, at least two TCM-based lifestyle elements are incorporated, such as "TCM-based exercise" and "TCM-based food therapy and/or nutritional advice" or "TCM-based psychological management" and "TCM-based sleep-wake management".

Six modules will be delivered on a consecutive six weeks as the subsequent sequences, week 1 for *nourishing the heart* 1, week 2 for *nourishing the heart* 2, week 3 for *nourishing according to time* 1, week 4 for *nourishing according to time* 2, week 5 for *nourishing the Qi* 1, and week 6 for *nourishing the Qi 2.*

Each module has the same structure consisting of introduction (10 to 15 minutes), pre-session knowledge test (5 to 10 minutes), the body of the session (around 60 minutes), checking, summary, question and answer (10 to 20 minutes), post-session knowledge test (5 to 10 minutes) and goal setting (5 to 10 minutes). The introduction will include warm-up, recap session, a review and discussion of the practical status of TCM lifestyle goals with the exception to the introduction of module 1 where a preliminary introduction regarding depression background globally and locally planning to be shared. Tasting herbal tea drink and providing herbal tea packet as souvenir will be provided at the first four sessions of the program. A 10-minute break will be provided in each session of the multicomponent TCM lifestyle medicine program for depression. The SMART (Specific, Measurable, Action-oriented, Realistic,

and Timed) traits will be applied while setting goals. During each session, interactive techniques, motivational interviewing strategies and teach-back method will be applied; and meanwhile, empathy and encouragement will be shown to participants.

In Table G1 and Tables G2 to G7, the whole structure of the multicomponent TCM lifestyle medicine program, as well as the structure and content of each session of the program are presented.

Table S12. The whole structure of multicomponent TCM lifestyle medicine program for depression.

Components	Sessions	Structures					
		**Introduction**		Body			
Nourishing the heart	*Nourishing the heart* 1	A preliminary introduction of depression background globally and locally	Pre-session knowledge test	Definition and function of health preservation and TCM health preservation, theoretical foundation of TCM health preservation, etiology, and pathogenesis as well as therapeutic strategies of TCM health preservation and emotion (depression), refined hobbies in *nourishing the heart* 1, practice *Baduajin*, provide syndrome differentiation-based diet suggestions for depression (1) and taste herbal tea drink (tangerine peel tea)	Summary and checking	Post-session knowledge test	Goal setting
	Nourishing the heart 2	Warm-up, recap session, a review and discussion of the practical status of TCM health preservation goals		Definition mental health care in *nourishing the heart,* the specific method of mental health care in *nourishing the heart (t*emperance method, catharsis method, *Qi* regulation, meditation as well as abstinence and essence conservation, clear heart, and calm the mind), practice meditation, provide syndrome differentiation-based diet suggestions for depression (2) taste herbal tea drink (rose drink)			
Nourishing according to time	*Nourishing according to time* 1			Concept and manifestation of *nourishing according to time*, concepts and principles of health-preserving daily schedule, "regulation before bedtime" (one sleep regulation method), introduce one acupoint (*Baihui*) that can improve sleep quality and relieve depression, learn and practice one acupoint (*Baihui*) that can improve sleep quality and relieve depression, provide syndrome differentiation-based diet suggestions for depression (3) (diet for better sleep), taste herbal tea drink (longan tea) (diet for better sleep)			
	Nourishing according to time 2			Introduction of "regulation at bedtime," "ten contraindications regarding sleep", two sleep regulation methods; principle of eating according to time and season, and introduce seasonal food; self-regulating sleep aids; introduce acupoints (*Yintang*, *Neiguan*, *Shenmen*) that can improve sleep quality and relieve depression, learn and practice one acupoint (*Yintang*, *Neiguan*, *Shenmen*) that can improve sleep quality and relieve depression; Provide syndrome differentiation-based diet suggestions for depression (4) and taste herbal tea (red date tea) for			

				alleviating depression (4) in class (diet for better sleep)			
Nourishing the Qi	*Nourishing the Qi* 1			Definition and function of *nourishing the Qi*, the relationship between "nourishment *Qi*" and "depression" in TCM, the relationship between Qigong and depression and the function of Qigong on depression, Experience nourishing *Qi* by learning and practicing *Baduanjin* in class, and introduce die related to TCM emotion and depression and nourishing the *Qi* 1			
	Nourishing the Qi 2			Diet suggestions related to TCM emotion and depression and nourishing the *Qi* 2; overview, function, and practical method of *Baduanjin*; experience nourishing the *Qi* by learning and practicing *Baduanjin* in class; and overall review of content of sessions of TCM health preservation program for depression			

Note. TCM = traditional Chinese medicine.

Table S13. The structure and content of nourishing the heart 1.

Session 1: *Nourishing the heart* 1	Objectives	Duration
Share depression experiences, depression background globally and in Hong Kong	To warm up and make participants feel peer atmosphere and realize not only me having depression	10 min.
Share expectations for program engagement and participation (e.g., expect to change)	To preliminarily understand participants' expectations of the program engagement and participation	
Pre-session 1 knowledge test	To test participants' understanding of knowledge regarding *nourishing the heart* 1 before session	
Introduce the definition and function of (TCM) health preservation	To master the definition and function of health preservation and TCM health preservation	25 min.
Introduce the theoretical foundation of TCM health preservation	To enrich the theoretical foundation of TCM health preservation of participants	
Introduce the etiology and pathogenesis of TCM health preservation and emotion (depression)	To deepen the understanding regarding the etiology and pathogenesis of TCM health preservation and emotion (depression)	
Introduce therapeutic strategies of TCM health preservation and emotion (depression)	To deepen therapeutic strategies of TCM health preservation and emotion (depression) understanding	
Introduce refined hobbies in *nourishing the heart* 1	To understand the definition of refined hobbies in *nourishing the heart* 1	
Break		10 min.
Practice *Baduanjin*, a method of qi regulation, win class with the program provider	To practice *Baduanjin* with the program provider	25 min.
Provide syndrome differentiation-based diet suggestions for depression (1) in class	To understand syndrome differentiation-based diet suggestions for depression (1) in class	20 min.
Taste herbal tea (tangerine peel tea) for alleviating depression (1) in class	To experience herbal tea for alleviating depression (1) in class	
Check	To confirm that participants understand TCM health preservation, as well as basic knowledge of TCM health preservation and depression	10 min.
	To confirm that participants understand definition of refined hobbies in *nourishing the heart* 1	
Post-session 1 knowledge test	To test participants' understanding of knowledge regarding nourishing the heart 1after	10 min.

	session	
Summary as well as question and answer section	To summarize the session and answer questions from participants	10 min.
Set SMART goals	To ensure that participants set TCM health preservation goals in the relevant logbook	
Reminder key points: continuous practice, at least once a day and at least two elements of the session each time	To ensure that participants understand practice expectations	

Note. SMART = specific, measurable, action-oriented, realistic, and timed; TCM = traditional Chinese medicine.

Table S14. The structure and content of nourishing the heart 2.

Session 1: *Nourishing the heart 2*	Objectives	Duration
Review content of session 1 (*nourishing the heart* 1)	To enhance participants' memory of the content of session 1<br>To promote participants' immersing in session atmosphere	10 min.
Share the completion status of TCM health preservation goals set in the logbook	To access the completion status of TCM health preservation goals set in the logbook	
Inquire questions encountered in TCM health preservation practice	To answer questions encountered in TCM health preservation practice	
Pre-session 2 knowledge test	To test participants' understanding of knowledge regarding *nourishing the heart* 2 before session	10 min.
Introduce the definition mental health care in *nourishing the heart*	To master the definition mental health care in *nourishing the heart*	25 min.
Describe the specific method of mental health care in *nourishing the heart*: Temperance method	To understand specific method of mental health care in *nourishing the heart*: Temperance method	
Describe the specific method of mental health care in *nourishing the heart*: Catharsis method	To understand specific method of mental health care in *nourishing the heart*: Catharsis method	
Describe the specific method of mental health care in *nourishing the heart*: Abstinence and essence conservation, clear heart, and calm the mind	To understand specific method of mental health care in *nourishing the heart*: Abstinence and essence conservation, clear heart, and calm the mind	
Describe the specific method of mental health care in *nourishing the heart*: *Qi* regulation	To understand specific method of mental health care in *nourishing the heart*: Qi regulation	
Break		10 min.
Describe the specific method of mental health care in *nourishing the heart*: Meditation	To understand specific method of mental health care in *nourishing the heart*: Meditation	10 min.
Practice meditation, a method of mental health care in class	To master the practice method of meditation	15 min.
Provide syndrome differentiation-based diet suggestions for depression (2) in class	To understand syndrome differentiation-based diet suggestions for depression (2) in class	15 min.
Taste herbal tea (rose drink) for alleviating depression (2) in class	To experience herbal tea for alleviating depression (2) in class	
Check	To ensure participants understand mental health care in *nourishing the heart*	10 min.
	To ensure participants master at least two methods in mental health care in *nourishing the heart*	
Post-session 2 knowledge test	To test participants' understanding of knowledge regarding *nourishing the heart* 2 after	5 min.

	session	
Summary as well as question and answer section	To summarize the session and answer questions from participants	10 min.
Set SMART goals	To ensure that participants set TCM health preservation goals in the relevant logbook	
Reminder key points: continuous practice, at least once a day and at least two elements of the session each time	To ensure that participants understand practice expectations	

Note. SMART = specific, measurable, action-oriented, realistic, and timed; TCM = traditional Chinese medicine.

Table S15. The structure and content of nourishing according to time 1.

Session 3: *Nourishing according to time* 1	Objectives	Duration
Review content of session 2 (*nourishing the heart* 2)	To enhance participants' memory of the content of session 2<br>To promote participants' immersing in session atmosphere	15 min.
Share the completion status of TCM health preservation goals set in the logbook	To access the completion status of TCM health preservation goals set in the logbook	
Inquire questions encountered in TCM health preservation practice	To answer questions encountered in TCM health preservation practice	
Pre-session 3 knowledge test	To test participants' understanding of *nourishing according to time* 1 before session	5 min.
Introduce the concept and manifestation of *nourishing according to time*	To understand the concept and manifestation of *nourishing according to time*	30 min.
Introduce the concepts and principles of health-preserving daily schedule	To master the concepts and principles of health-preserving daily schedule	
Introduce "regulation before bedtime", one of the sleep regulation methods	To understand "regulation before bedtime", one of the sleep regulation methods	
Introduce acupoints that can improve sleep quality and relieve depression in class (1): Baihui (DU20)	To understand acupoints that can improve sleep quality and relieve depression in class 1: Baihui (DU20)	
Break		10 min.
Learn and practice acupoints that can improve sleep quality and relieve depression in class (1): Baihui (DU20)	To master one acupoint that can improve sleep quality and relieve depression in class 1: Baihui (DU20)	10 min.
Provide syndrome differentiation-based depression diet suggestions (3) in class (diet for sleep)	To understand syndrome differentiation-based diet suggestions for depression (3) in class	20 min.
Taste herbal tea (longan tea) for alleviating depression (3) in class (diet for better sleep)	To experience herbal tea for alleviating depression (3) in class	
Check	To ensure that participants master the concept of health-preserving daily schedule and sleep regulation methods	15 min.
	To ensure participants master one acupoint that can improve sleep quality and relieve depression	
Post-session 3 knowledge test	To test participants' understanding of *nourishing according to time* 1 after session	10 min.
Summary as well as question and answer section	To summarize the session and answer questions from participants	5 min.
Set SMART goals	To ensure that participants set TCM health preservation goals in the relevant logbook	

Reminder key points: continuous practice, at least once a day and at least two elements of the session each time	To ensure that participants understand practice expectations	

Note. SMART = specific, measurable, action-oriented, realistic, and timed; TCM = traditional Chinese medicine.

Table S16. The structure and content of nourishing according to time 2.

Session 3: *Nourishing according to time* 2	Objectives	Duration
Review content of session 3 (n*ourishing according to time* 1)	To enhance participants' memory of the content of session 3; To promote participants' immersing in session atmosphere	15 min.
Share the completion status of TCM health preservation goals set in the logbook	To access the completion status of TCM health preservation goals set in the logbook	
Inquire questions encountered in TCM health preservation practice	To answer questions encountered in TCM health preservation practice	
Inquire the completion status of pressing acupoint that can improve sleep quality and relieve depression (Baihui)	To assess the completion status of pressing acupoint that can improve sleep quality and relieve depression (Baihui); To give affirmation and encouragement of participants who completed and thus increase their self-efficacy	
Pre-session 4 knowledge test	To test participants' understanding of knowledge regarding *nourishing according to time* 2 before session	5 min.
Introduce "regulation at bedtime," and "ten contraindications regarding sleep", two sleep regulation methods	To understand "regulation at bedtime," and "ten contraindications regarding sleep", two sleep regulation methods	25 min.
Introduce the principle of eating according to time and season, and introduce seasonal food	To understand the principle of eating according to time and season, and introduce seasonal food	
Introduce self-regulating sleep aids (meditation, self-hypnosis to fall asleep, tranquility)	To understand self-regulating sleep aids (meditation, self-hypnosis to fall asleep, tranquility)	
Learn and practice acupoints that can improve sleep quality and relieve depression in class (2): Yintang (EX－HN3)	To master acupoints that can improve sleep quality and relieve depression in class (2): Yintang (EX－HN3)	
Break		10 min.
Learn and practice acupoints that can improve sleep quality and relieve depression in class (2): Neiguan (PC6)	To master acupoints that can improve sleep quality and relieve depression in class (2): Neiguan (PC6)	10 min.
Learn and practice acupoints that can improve sleep quality and relieve depression in class (2): Shenmen (HT7)	To master acupoints that can improve sleep quality and relieve depression in class (2): Shenmen (HT7)	10 min.
Provide syndrome differentiation-based diet suggestions for depression (4) in class (diet for better sleep)	To undserstand syndrome differentiation-based diet suggestions for depression (4) in class (diet for better sleep)	15 min.

Taste herbal tea (red date tea) for alleviating depression (4) in class (diet for better sleep)	To experience herbal tea for alleviating depression (4) in class	
Check	To ensure participants master the concept of sleep regulation methods and self-regulating sleep aids	15 min.
	To ensure that participants master one acupoint that can improve sleep quality and relieve depression	
Post-session 4 knowledge test	To test participants' understanding of knowledge regarding *nourishing according to time* 2 after session	5 min.
Summary as well as question and answer section	To summarize the session and answer questions from participants	10 min.
Set SMART goals	To ensure that participants set TCM health preservation goals in the relevant logbook	
Reminder key points: continuous practice, at least once a day and at least two elements of the session each time	To ensure that participants understand practice expectations	

Note. SMART = specific, measurable, action-oriented, realistic, and timed; TCM = traditional Chinese medicine.

Table S17. The structure and content of nourishing the Qi 1.

Session 5: Nourishing the *Qi* 1	Objectives	Duration
Review content of session 4 (n*ourishing according to time* 2)	To enhance participants' memory of the content of session 3<br>To promote participants' immersing in session atmosphere	15 min.
Share the completion status of TCM health preservation goals set in the logbook	To access the completion status of TCM health preservation goals set in the logbook	
Inquire questions encountered in TCM health preservation practice	To answer questions encountered in TCM health preservation practice	
Inquire the completion status of pressing acupoint that can improve sleep quality and relieve depression (Yintang, Neiguan, Shenmen)	To assess the completion status of pressing acupoint that can improve sleep quality and relieve depression (Yintang, Neiguan, Shenmen)<br>To give affirmation and encouragement of participants who completed and thus increase their self-efficacy	
Pre-session 5 knowledge test	To test participants' understanding of knowledge regarding *nourishing the Qi* 1 before session	10 min.
Introduce the definition and function of *nourishing the Qi*	To understand the definition and function of *nourishing the Qi*	20 min.
Introduce the relationship between "nourishment *Qi*" and "depression" in TCM ("adjustment of body," "regulation of breathing," and "regulation of mental activities")	To understand the relationship between "nourishment *Qi*" and "depression" in TCM ("adjustment of body," "regulation of breathing," and "regulation of mental activities")	
Introduce the relationship between Qigong and depression and the function of Qigong on depression	To understand the relationship between Qigong and depression and the function of Qigong on depression	
Break		10 min.
Experience nourishing the *Qi* by learning and practicing *Baduanjin* in class	To consolidate and master *Baduanjin*	25 min.
Introduce diet suggestions related to TCM emotion and depression and nourishing the *Qi* (1)	To understand diet suggestions related to TCM emotion and depression and nourishing the *Qi* (1)	10 min.
Check	To ensure that participants understand the definition and function of *nourishing the Qi*, and the relationship between nourishing the *Qi*	15 min.
	To ensure that participants understand the relationship between Qigong and depression and the function of Qigong on depression	
Post-session 5 knowledge test	To test participants' understanding of knowledge regarding *nourishing the Qi* 1 after session	5 min.

Summary as well as question and answer section	To summarize the session and answer questions from participants	10 min.
Set SMART goals	To ensure that participants set TCM health preservation goals in the relevant logbook	
Reminder key points: continuous practice, at least once a day and at least two elements of the session each time	To ensure that participants understand practice expectations	

Note. SMART = specific, measurable, action-oriented, realistic, and timed; TCM = traditional Chinese medicine.

Table S18. The structure and content of nourishing the Qi 2.

Session 6: Nourishing the *Qi* 2	Objectives	Duration
Review content of session 4 (*nourishing according to time* 2)	To enhance participants' memory of the content of session 3	15 min.
Share the completion status of TCM health preservation goals set in the logbook	To promote participants' immersing in session atmosphere	
Inquire questions encountered in TCM health preservation practice	To access the completion status of TCM health preservation goals set in the logbook	
Pre-session 6 knowledge test	To test participants' understanding of knowledge regarding *nourishing the Qi* 2 before session	5 min.
Introduce diet suggestions related to TCM emotion and depression and nourishing the *Qi* (2)	To understand diet suggestions related to TCM emotion and depression and nourishing the *Qi* (2)	20 min.
Introduce *Baduanjin*, a method for nourishing the Qi, including overview, function, and practical method	To understand *Baduanjin*, a method for nourishing the Qi, including overview, function, and practical method	
Break		10 min.
Experience nourishing the Qi by learning and practicing *Baduanjin* in class	To consolidate and master *Baduanjin*	25 min.
Check	To ensure that participants understand diet suggestions related to TCM emotion and depression and nourishing the *Qi*	10 min.
	To master *Badunjin's* overview, function, practical method and practical process	
Overall review of content of sessions of TCM health preservation program for depression	To review and consolidate content of sessions of TCM health preservation program for depression	20 min.
Pre-session 6 knowledge test	To test participants' understanding of knowledge regarding *nourishing the Qi* 2 after session	5 min.
Summary as well as question and answer section	Summary as well as question and answer section	10 min.
Set SMART goals	Set SMART goals	
Reminder key points: continuous practice, at least once a day and at least two elements of the session each time	Reminder key points: continuous practice, at least once a day and at least two elements of the session each time	

Note. SMART = specific, measurable, action-oriented, realistic, and timed; TCM = traditional Chinese medicine.

4.3 Program materials

Program materials mainly include two parts, teaching related materials and assessment-related materials. Teaching related materials include teaching materials for sessions of the multicomponent TCM lifestyle medicine program for depression (slides and printed version), logbooks for the multicomponent TCM lifestyle medicine program for depression, and herbal tea packet. Assessment-related materials incorporate session knowledge test, session knowledge test, acceptability scale, or program participants'/researchers'/CMPs' assessment form. All those materials will be distributed to participants when they attend each session.

Figure S2. *Nourishing the heart 1.*

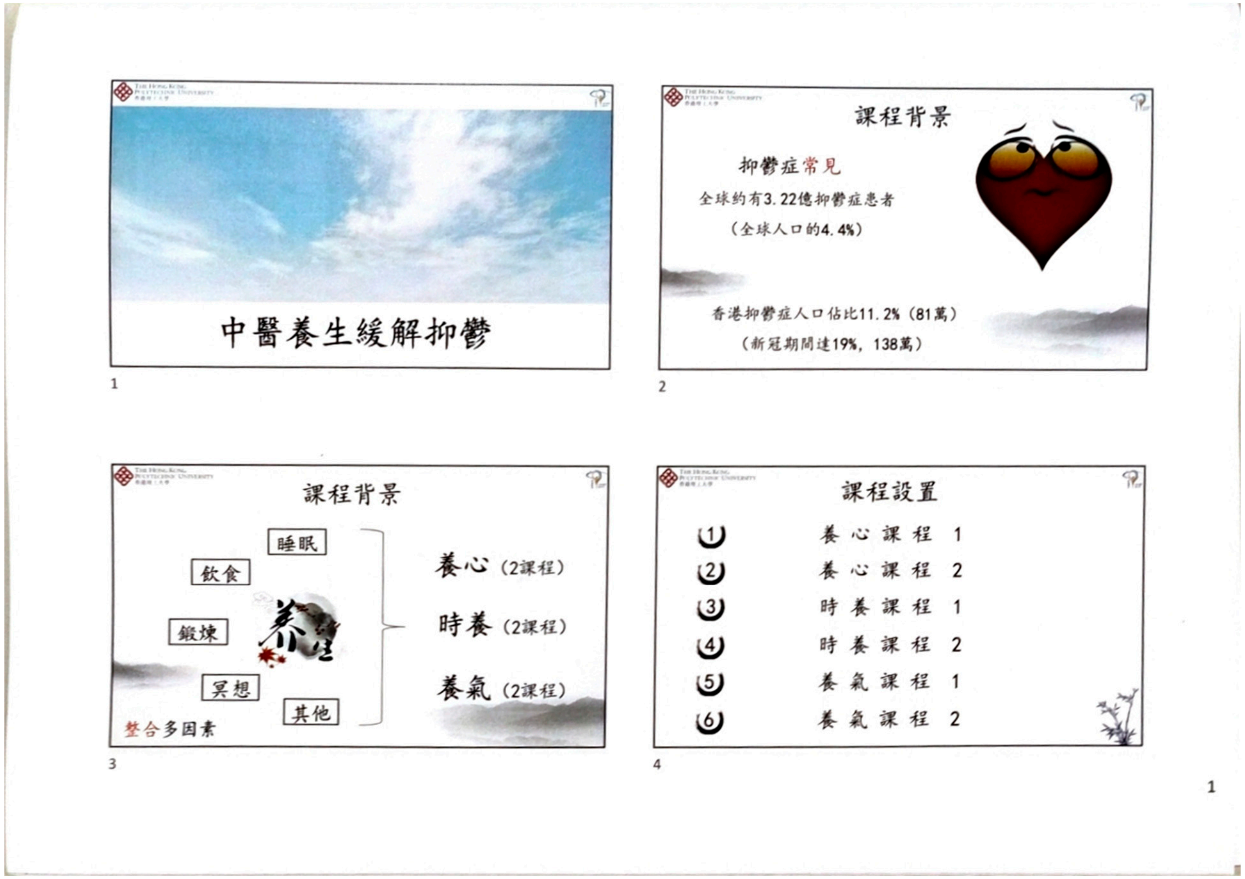

Figure S3. *Nourishing the heart 2.*

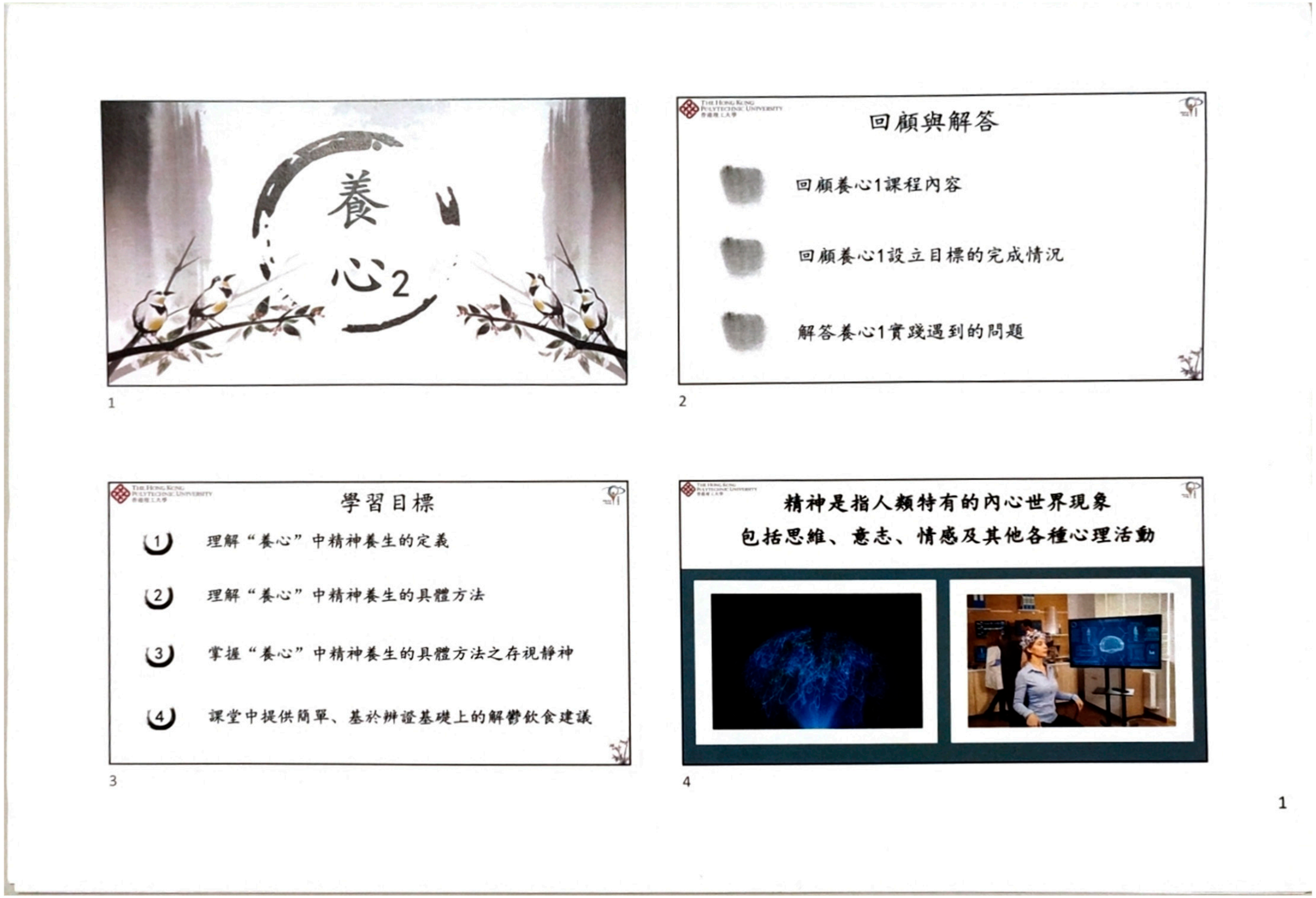

Figure S4. *Nourishing according to the time 1.*

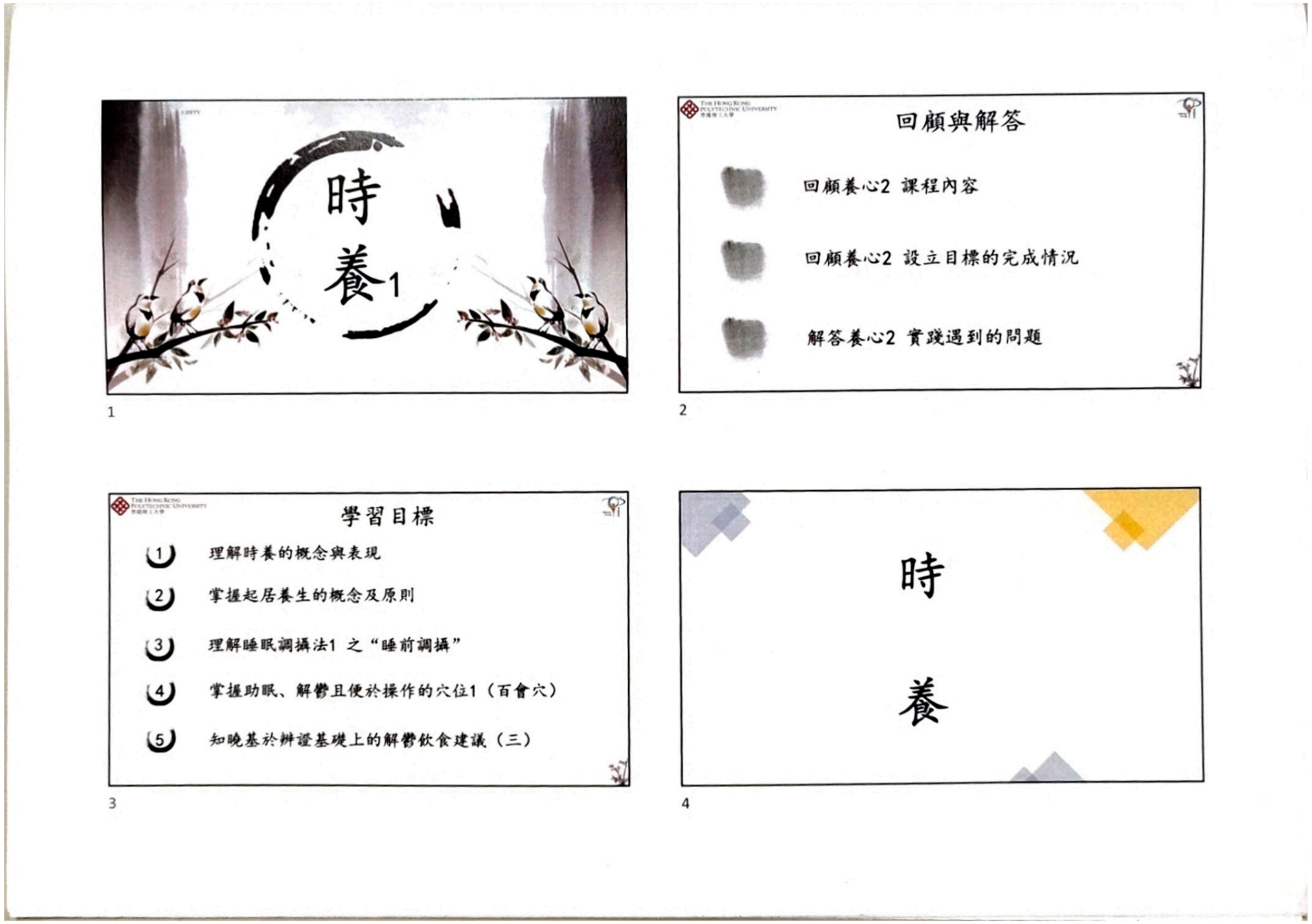

Figure S5. *Nourishing according to the time 2.*

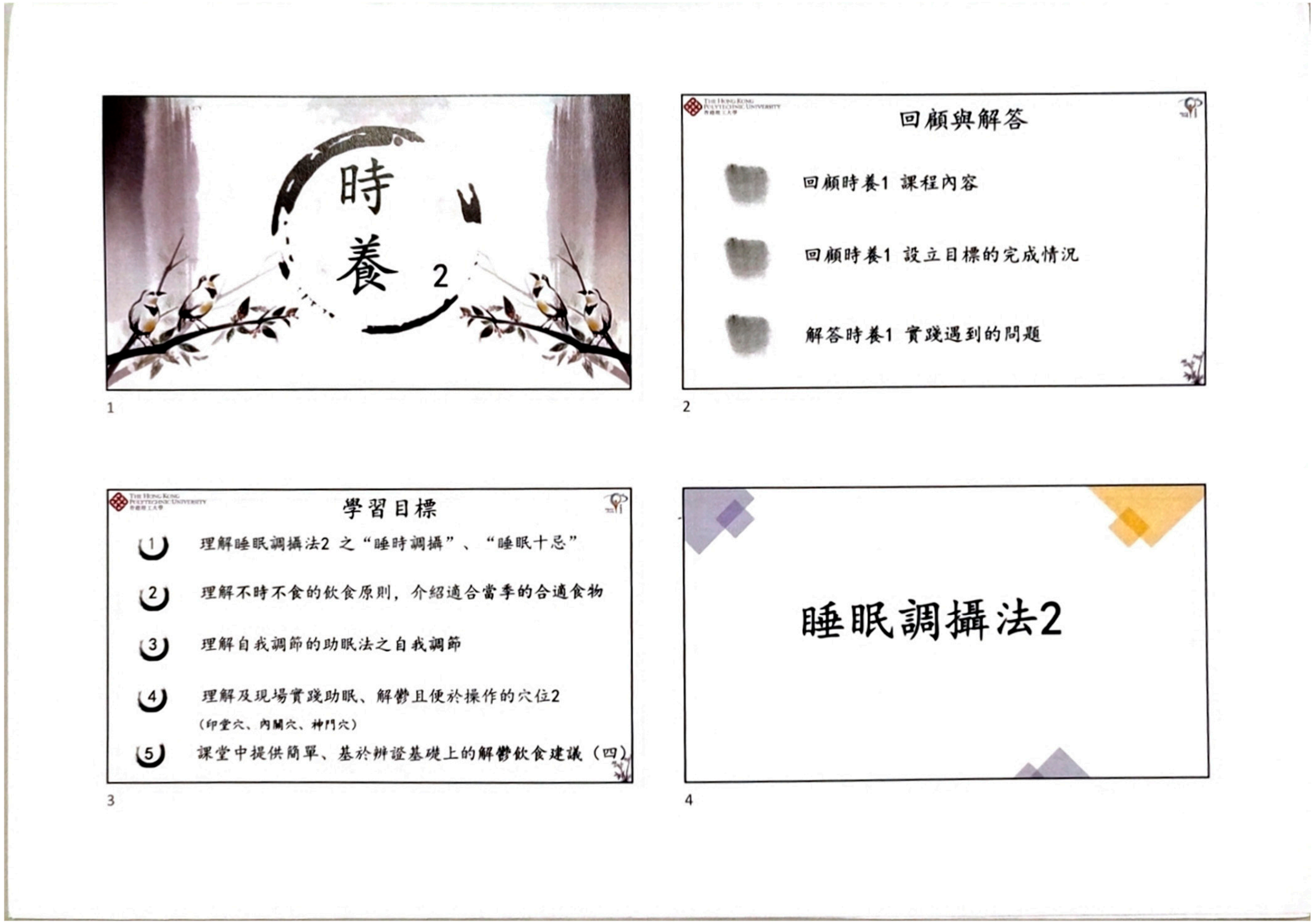

Figure S6. *Nourishing the Qi 1.*

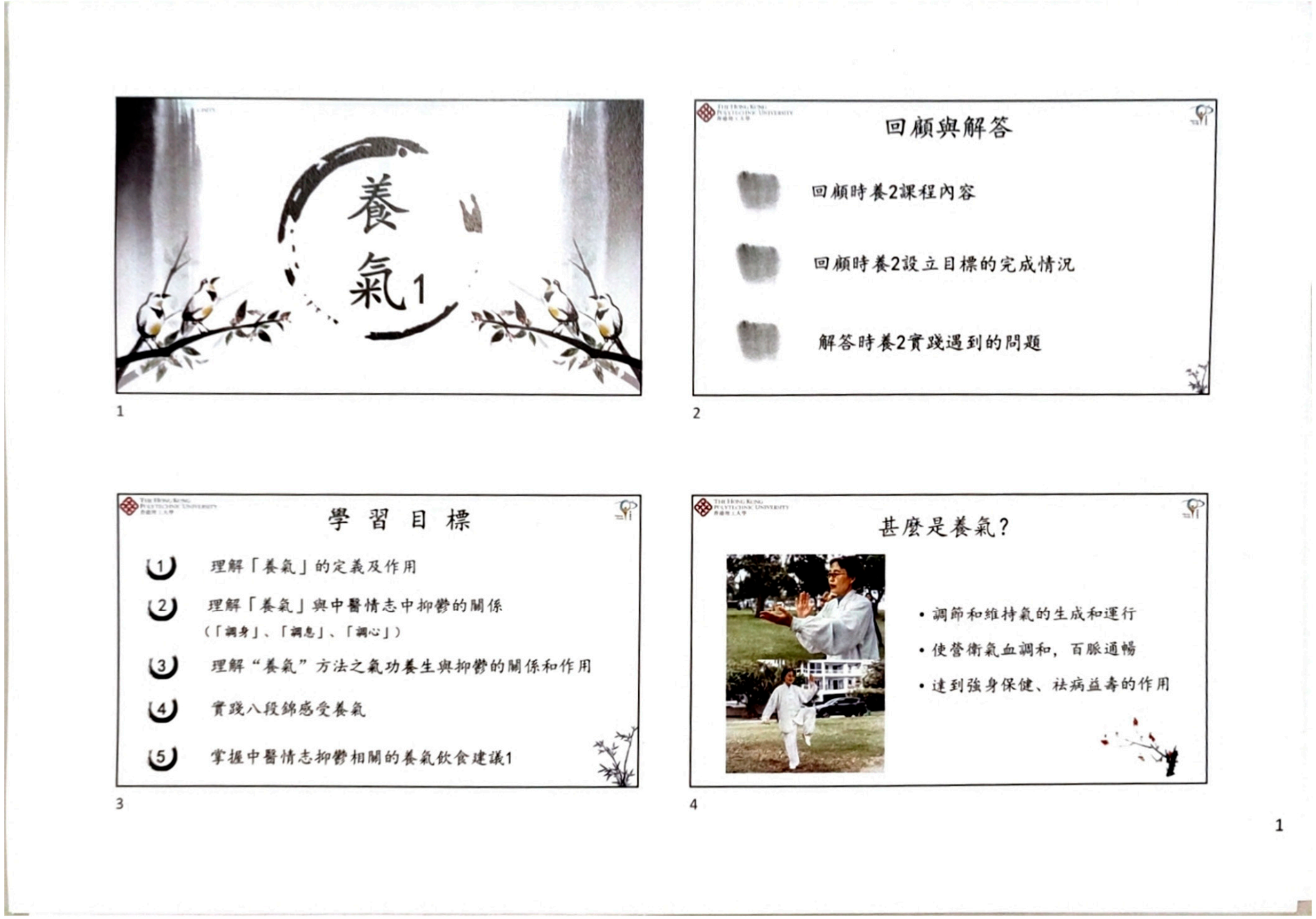

Figure S7. *Nourishing the Qi 2.*

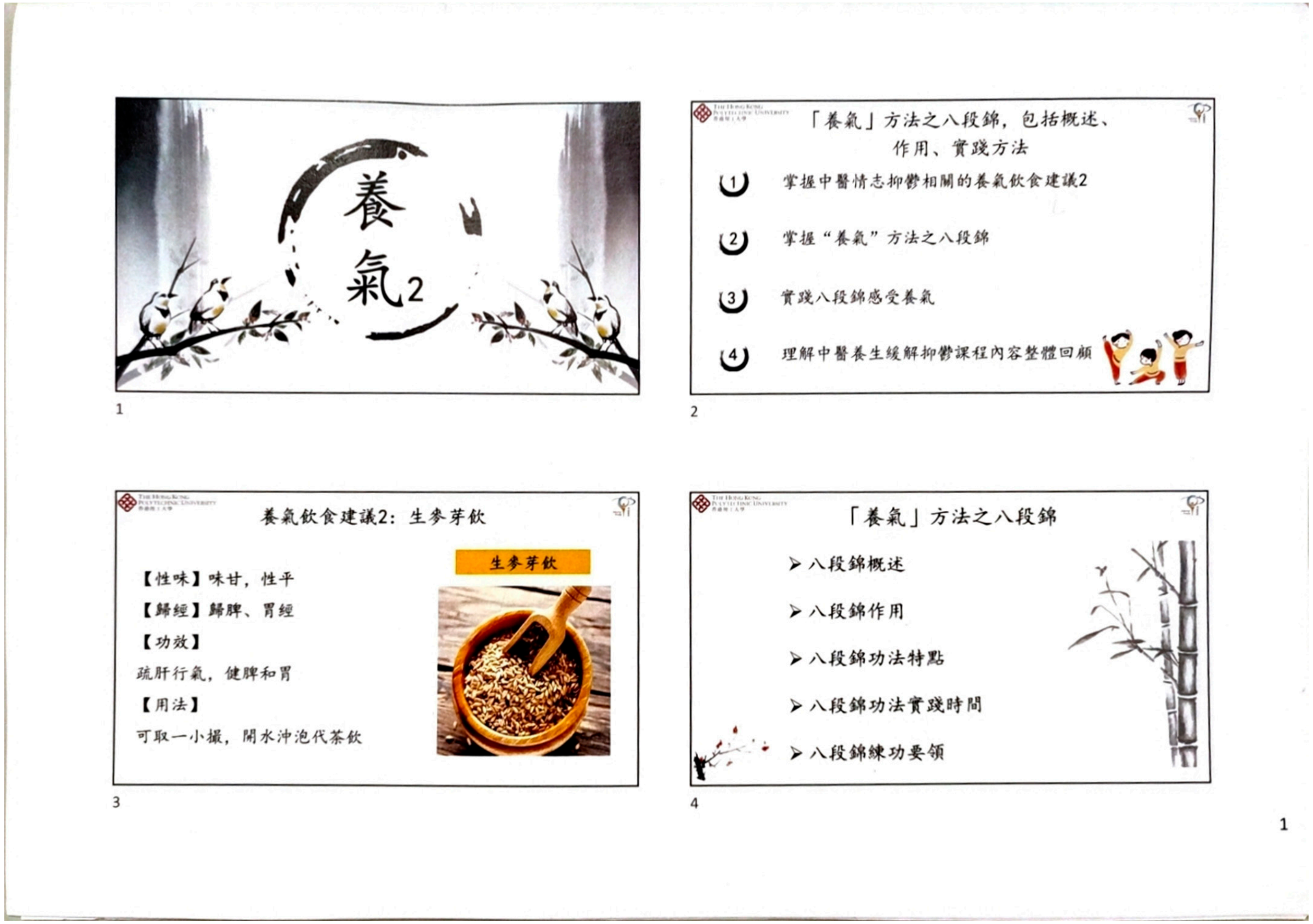

Table S19. Explanations and corresponding assumptions of conceptual framework for the multicomponent traditional Chinese medicine lifestyle medicine program.

Explanations		Assumptions	
1	The Traditional Chinese medicine health preservation program for depression targets Chinese adults aged 18–65 years with moderate depression in Hong Kong.	1	The number of Chinese adults suffering from depression in Hong Kong continues to increase, and targeting this population will meet those individuals' demand for finding a novel depression treatment approach that is context based, theory based, evidence-based, and less stigmatized.
2	The program involving integrated Traditional Chinese medicine-based lifestyle components (Traditional Chinese medicine health preservation) aims to reduce depression and improve other health-related outcomes among Chinese adults with moderate depression in Hong Kong.	2	Increasing the knowledge and skills needed in Traditional Chinese medicine health preservation will lead to decreased depression and improved health-related outcomes among Chinese adults with moderate depression in Hong Kong
3	Activities in the Traditional Chinese medicine health preservation program include six two-hour weekly group sessions, Traditional Chinese medicin health preservation goal setting, distribution of printed teaching materials, allocation of logbooks, tasting herbal tea drink, and providing herbal tea packet as souvenir.	3	By providing activities in the Traditional Chinese medicine health preservation program, participants will have an increased chance to increase their knowledge and skills of Traditional Chinese medicine health preservation and have an improved possibility to adhere to practice Traditional Chinese medicine health preservation.
4	Stakeholders involved in the program include a clinical psychologist, a psychiatrist, a mental health nurse, four Hong Kong Chiense medicine practitioners, project funders, and Hong Kong adults with depression.	4	Collaborating with various stakeholders will foster support, community engagement, and sustainability for the program.
5	The program operates on the developed program theory and will	5	A program with integrated Traditional Chinese medicine -based lifestyle components will yield

	lead to depression reduction and other health-related outcomes improvement.		positive outcomes.
6	The program allocates resources for hiring qualified Chinese Medicine practitioners; designing, making and binding teaching materials and Traditional Chinese medicine health preservation logbooks; and preparing herbal tea drink in sessions and providing herbal tea packet as souvenir.	6	Adequate resources and strategic investments are essential to implementing effective programs and achieving program objectives.
7	The program incorporates regular monitoring and evaluation mechanisms to measure progress, assess outcomes, and make data-driven decisions.	7	Continuous monitoring and evaluation are important for assessing the feasibility, acceptance, and effects of the program, identifying areas for improvement, and ensuring accountability to stakeholders.
8	The program acknowledges local contexts, cultural diversity, and community preferences in its design and implementation.	8	Respecting values, cultural norms, and community contexts improves program relevance, impact, and acceptance.
9	The program recognizes potential external factors that may impact its implementation and outcomes.	9	Anticipating external factors and risks allows the program to adapt, mitigate challenges, and maintain its relevance and effectiveness over time.
